# Construction of Multifunctional Conductive Carbon-Based Cathode Additives for Boosting Li_6_PS_5_Cl-Based All-Solid-State Lithium Batteries

**DOI:** 10.1007/s40820-025-01667-7

**Published:** 2025-02-11

**Authors:** Xin Gao, Ya Chen, Zheng Zhen, Lifeng Cui, Ling Huang, Xiao Chen, Jiayi Chen, Xiaodong Chen, Duu-Jong Lee, Guoxiu Wang

**Affiliations:** 1 College of Smart Energy, Shanghai Jiao Tong University, Shanghai, 200240 People’s Republic of China; 2 Smart Materials for Architecture Research Lab, Innovation Center of Yangtze River Delta, Zhejiang University, Jiashan, 314100 People’s Republic of China; 3 Department of Mechanical Engineering, City University of Hong Kong, Tat Chee Avenue, Kowloon, 999077 People’s Republic of China; 4 Centre for Clean Energy Technology, School of Mathematical and Physical Science, Faculty of Science, University of Technology Sydney, Sydney, 2007 Australia; 5 School of Physical Science and Technology, Shanghai Tech University, Shanghai, 201210 People’s Republic of China

**Keywords:** Multifunctional conductive-carbon additives, Mo-Ni@NPCs, Sulfide solid electrolytes, Cathodes interfaces stabilities, All-solid-state lithium batteries

## Abstract

The electrochemical performance of all-solid-state lithium batteries (ASSLBs) can be prominently enhanced by minimizing the detrimental degradation of solid electrolytes through their undesirable side reactions with the conductive carbon additives (CCAs) inside the composite cathodes. Herein, the well-defined Mo_3_Ni_3_N nanosheets embedded onto the N-doped porous carbons (NPCs) substrate are successfully synthesized (Mo-Ni@NPCs) as CCAs inside LiCoO_2_ for Li_6_PSC_5_Cl (LPSCl)-based ASSLBs. This nano-composite not only makes it difficult for hydroxide groups (–OH) to survive on the surface but also allows the in situ surface reconstruction to generate the ultra-stable MoS_2_-Mo_3_Ni_3_N heterostructures after the initial cycling stage. These can effectively prevent the occurrence of OH-induced LPSC decomposition reaction from producing harmful insulating sulfates, as well as simultaneously constructing the highly-efficient electrons/ions dual-migration pathways at the cathode interfaces to facilitate the improvement of both electrons and Li^+^ ions conductivities in ASSLBs. With this approach, fine-tuned Mo-Ni@NPCs can deliver extremely outstanding performance, including an ultra-high first discharge-specific capacity of 148.61 mAh g^−1^ (0.1C), a high Coulombic efficiency (94.01%), and a capacity retention rate after 1000 cycles still attain as high as 90.62%. This work provides a brand-new approach of “conversion-protection” strategy to overcome the drawbacks of composite cathodes interfaces instability and further promotes the commercialization of ASSLBs.

**Supplementary Information:**

The online version contains supplementary material available at 10.1007/s40820-025-01667-7.

## Highlights

- This work provides a brand-new approach to the “conversion-protection” strategy to overcome the drawbacks of composite cathode interfaces.
- The Mo_3_Ni_3_N not only makes it difficult for hydroxide groups (-OH) to survive on the surface but also allows the in situ surface reconstruction to generate the ultra-stable MoS_2_-Mo_3_Ni_3_N heterostructures after the initial cycling stage.
- The Mo-Ni@NPCs/LCO/LPSC-based ASSLBs achieve high-capacity retention (90.62%) and excellent cycle life (1000 cycles).

**Supplementary Information:**

The online version contains supplementary material available at 10.1007/s40820-025-01667-7.

## Graphical abstract

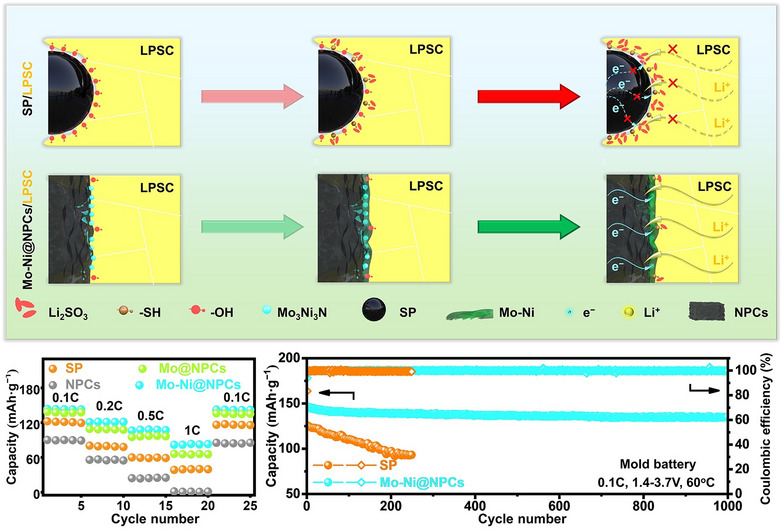

## Introduction

All-solid-state lithium batteries (ASSLBs) have been acknowledged as promising substitutes for conventional Li-ion batteries (LIBs) due to their high energy density and guaranteed intrinsic safety [1–3]. This considerably associates with the fact that they perfectly inherit the comprehensive advantages of high theoretical specific capacity (3800 mAh g^−1^), low electrochemical reduction potential (− 3.04 V vs. SHE) and low mass density (0.53 g cm^−3^) of Li anodes, as well as low-level feasibility of combustibility, leakage and vaporization of inorganic solid electrolytes (SEs) [4, 5]. As a key component of ASSLBs, the exploration of preferable SEs has become the prerequisite and foundation for acquiring high-performance ASSLBs [6]. Among the various SEs systems being extensively researched, sulfide-solid electrolytes (SSEs), including Li_6_PS_5_Cl (LPSC) [7], Li_3_PS_4_ [8], Li_10_GeP_2_S_12_ [9], Li_2_S-P_2_S_5_ [10] and Li_9.54_Si_1.74_(P_0.9_Sb_0.1_)_1.44_S_11.7_Cl_0.3_ [11] have been swimmingly implemented due to their superior Li^+^ ions conductivity (> 1.0 mS cm^−1^ at ambient temperature) that can be comparable to or outperform liquid electrolytes (LEs), which give impetus for the large-scale application of ASSLBs.

Notwithstanding these benefits, there still exist some intrinsic issues that need to be addressed regarding the integration of SSEs into ASSLBs systems, especially the interface instability problems inside composite cathodes, which constitutes a key technological bottleneck for boosting battery performance [12]. More concretely, in practical assembly of ASSLBs, the composite cathodes are typically consisted of active materials (AMs), SSEs, and conductive carbon additives (CCAs), whose electrochemical properties are completely influenced by physicochemical properties of these constituents [13]. Therein, the CCAs are used to build up the sprawling electron migration pathways among the AMs throughout the composite cathodes [14]. Nevertheless, the degradative side reactions at the CCAs/SSEs interface are extremely prone to happen due to the intrinsically chemical and electrochemical instabilities of SSEs, where they are more thermodynamically reactive with the oxygen-containing functional groups over the CCAs [15]. These side reactions will accelerate the decomposition of SSEs and meanwhile generate the detrimental sulfates, which not only block Li^+^ ions and electron transport but may also trigger the serious contact failure at the cathode interfaces. These actions deteriorate the capacity and cycling performance of ASSLBs [16]. What’s more, when incorporating the CCAs into the cathode materials, the percolated SSE networks may not fully interconnect, thereby restraining the Li^+^ ions transport [17]. Therefore, it is of great importance to have effective strategies for the resolution of the above-mentioned concerns brought on by the introduction of suitable CCAs inside composite cathode for ASSLBs.

As of now, the considerable engineering approaches have been executed to optimize the structural functionalities of CCAs, including the architecture of encapsulation layer [18], heteroatoms doping [13], nanostructure tailoring [19], and hybriding [20] for avoiding the parasitic side reactions at CCAs/SSEs interface. Among these approaches, hybridizing the other appropriate phases into CCAs has typically been used to insert additional functionalities to compensate for functional deficiencies in cathodes. This has been considered as one of the most feasible methods to suppressing SSEs degradation and furnishing the Li^+^ ions/electrons transportation pathways, thus giving rise to the increased intrinsic performance [21]. In this regard, designing the endogenous heterojunctions of MoS_2_ coupled with Mo-based mono/bi-metallic nitrides, such as MoS_2_-MoN and MoS_2_-Mo_3_Ni_3_N, can be regarded as the promising candidates for introducing phases with their high structural stability, adjustable electronic structure, and especially sufficient active sites for rapid ion/electron transport and desirable Li^+^ ions storage capability [22, 23]. Apart from these promotions, the compositing of these heterojunctions with CCAs would upgrade the electron conductivity and weaken the interaction with SSEs as well as simultaneously further anchor, decentralize, and assemble the heterojunctions sites themselves, thus contributing to the enhancement of integrated electron transfer ability, desirable prohibition of SSEs degradation, reinforcement of structural ability, and increment in the quantity of accessible Li^+^ ions transformation pathways [24]. Unfortunately, although this category of ultrafine heterojunctions added has been demonstrated to be more beneficial for ameliorating the unstable CCAs/SSEs interface, it has rarely appeared in previous investigation efforts on composite cathodes for ASSLBs [13]. Therefore, it is becoming strategic and timely to fabricate the composites of these well-defined heterojunctions with CCAs to comprehensively eliminate CCAs/SSEs interface issues in LPSC-based ASSLBs, even though they still remain a significant challenge.

**Fig. 1:**
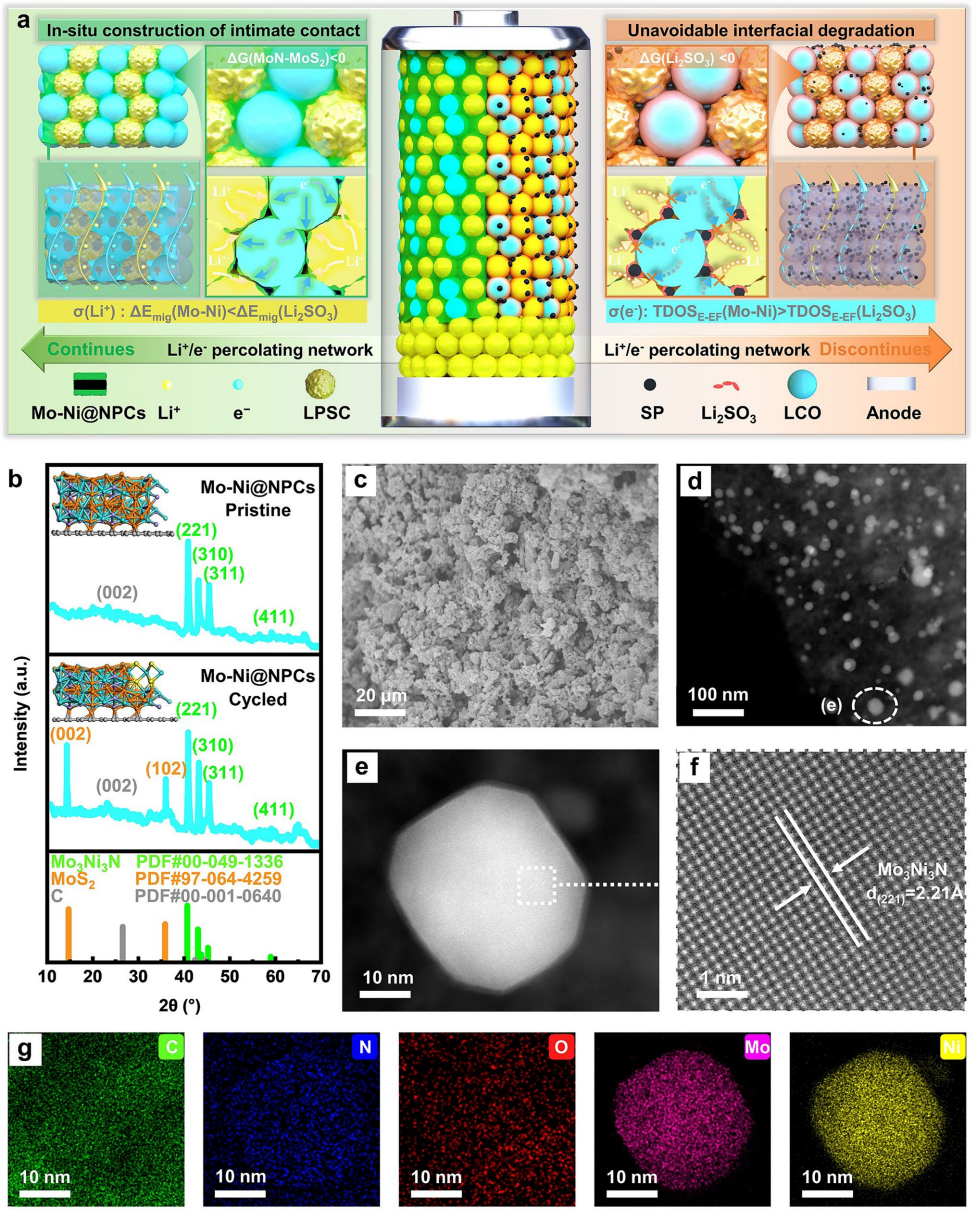
**a** Schematic diagram of the mechanism of Mo-Ni@NPCs at the cathode interfaces. **b** XRD patterns of Mo-Ni@NPCs (before and after 100 cycles). **c** SEM image, **d-f** HAADF-STEM images and **g** EDS elemental mapping images of Mo-Ni@NPCs

Enlightened by the aforementioned analysis of strategies, we accurately synthesize a well-defined Mo_3_Ni_3_N embedded onto the N-doped porous carbons (NPCs) substrates (Mo-Ni@NPCs) by an association of wet-impregnation treatment of Ni, Mo/cyclodextrin-based nanosponges (CDNS) precursor, following by thermal annealing, which initiate its surface to undergo the reconstruction and in situ form the ultra-stable MoS_2_-Mo_3_Ni_3_N heterostructures over the NPCs near the SSEs regions after cycling. These multifunctional CCAs can entirely inherit the respective chemical properties of these constituents well, and can be subsequently used to avoid the occurrence of SSEs degradation reaction which cause the interfaces contact failure and instead construct the effective Li^+^ ions/electrons transformation pathways to facilitate their corresponding migration across the composite cathode interfaces (Fig. 1a). Performance evaluation indicates that ASSLBs using this cathode additive system deliver an ultra-high first discharge specific capacity of 148.61 mAh g^−1^ at a current density of 0.1C with an exceptional Coulombic efficiency of 94.01%. It also presents prominent stability with a capacity retention rate of 90.62% after 1000 cycles, and promising commercial prospects with its ultra-high first discharge specific capacity of 127.55 mAh g^−1^ under high areal capacity conditions of 3.0 mAh cm^−2^. This contribution paves up a novel direction for the preparation of CCAs cathode materials, and effectively boosts the commercialization prospects of ASSLBs.

## Experimental Section

### Materials

The span60 (C_24_H_46_O_6_, CP), tween20 (C_26_H_50_O_10_,CP), melamine (C_3_H_6_N_6_, CP, 99.0%), β-cyclodextrin (C_42_H_70_O_35_, LC, 98.0%) and epichlorohydrin (C_3_H_5_ClO, AR, 99.5%) are provided by Sinopharm Group Co., Ltd. anhydrous ethanol (C_2_H_5_OH, AR, 99.5%), kerosene (C_15_H_32_, AR), ultrapure water (H_2_O, electric conductivity (20 °C) = 2.10 μS cm^−1^), ammonium molybdate tetrahydrate (H_24_Mo_7_N_6_O_24_·4H_2_O, AR, 99.0%) and nickel chloride hexahydrate (NiCl_2_·6H_2_O, AR, 99.9%) are provided by Aladdin Reagent Co., Ltd. Li_6_PS_5_Cl (LPSC) is self-made based on the previous reports [7]. LiCoO_2_ (LCO) is provided by XTC New Energy Materials (Xiamen) Co., Ltd. All the chemicals are used as received without further purification.

### Synthesis of Cyclodextrin-Based Nanosponges Materials

The nanosponge is prepared by inverse emulsion polymerization. In the 200 mL of kerosene, span60 and tween20 are sequentially added to form a continuous oil phase. In a separate container containing 50 mL of H_2_O, β-cyclodextrin, epichlorohydrin, and melamine are sequentially added, and the mixture is stirred at 300 r min^−1^ for 30 min at 30 °C to obtain a dispersed phase. The dispersed phase is then added to the continuous oil phase at a rate of 5 mL min^−1^, and the temperature is raised to 70 °C for 3 h to synthesize the nanosponge.

### Synthesis of NPCs, Mo@NCPs, and Ni-Mo@NPCs

#### Synthesis of NPCs

The NPCs are synthesized by carbonizing in a tube furnace under a nitrogen atmosphere. Typically, 20 g of nanosponges are placed into a tube furnace filled with nitrogen and carbonized at 700 °C for 4 h to obtain NPCs.

#### Synthesis of Mo@NPCs

The Mo@NPCs are synthesized by impregnating nanosponges with a metal salt solution and carbonizing them in a tube furnace under a nitrogen atmosphere. Specifically, 20 g of nanosponges are immersed in 100 mL solution containing 0.005 mol of H_24_Mo_7_N_6_O_24_·4H_2_O of solvent for 24 h. Subsequently, the solvent is removed by freeze-drying in the lyophilizer to ensure adequate attachment of metal salt molecules to the polymer surface (− 60 °C, 9 Pa). Finally, the samples are placed into a tube furnace filled with nitrogen and carbonized at 700 °C for 4 h to obtain Mo@NPCs.

#### Synthesis of Mo-Ni@NPCs

The Mo@NPCs are synthesized by impregnating nanosponges with a metal salt solution and carbonizing them in a tube furnace under a nitrogen atmosphere. Specifically, 20 g of nanosponges are immersed in 100 mL solution containing 0.005 mol of H_24_Mo_7_N_6_O_24_·4H_2_O and 0.05 mol of NiCl_2_·6H_2_O of solvent for 24 h. Subsequently, the solvent is removed by freeze-drying in the lyophilizer to ensure adequate attachment of metal salt molecules to the polymer surface (−60 °C, 9 Pa). Finally, the samples are placed into a tube furnace filled with nitrogen and carbonized at 700 °C for 4 h to obtain Mo-Ni@NPCs. More details can be found in Supporting Information.

## Results and Discussion

### Establishments and Characterization of Mo-Ni@NPCs

The synthesized procedures of Mo-Ni@NPCs, Mo@NPCs and NPCs through polymerization, wet-impregnation and thermal treatment are depicted in Figs. S1–S3. The X-ray diffraction (XRD) measurements are employed for the structural characterization of these samples, as shown in Figs. 1b and S4. All the samples appear an identical characteristic peak at 26.54°, attributed to C (002) crystal plane (PDF#00–001-0640), Besides this regularity, other distinctive characteristic peaks of Mo-Ni@NPCs and Mo@NPCs can be observed at 40.70°, 43.01°, 45.23°, 58.97° and 31.65°, 36.05°, and 48.73°, which corresponds to (211), (310), and (311) crystal planes of Mo_3_Ni_3_N (PDF#00–049-1336) and (002), (200), and (202) crystal planes of MoN (PDF#01–074-7404). These results indicate these samples are successfully synthesized as expected under our experimental conditions, also further confirmed by XPS results (Fig. S5). The microscopic morphologies and structural features of Mo-Ni@NPCs, Mo@NPCs, NPCs and Super-P (SP) samples are determined by SEM and TEM characterizations. As shown in Figs. 1c and S6, the microscopic morphologies of SP consist primarily of uniformly sized micro-scale spherical particles, whereas for NPCs, Mo-Ni@NPCs, and Mo@NPCs samples, their morphologies remain similar and behave as irregular aggregates of microspheres. In addition, the corresponding HADDF-STEM, HRTEM, SAED, and EDS element mapping images of Mo-Ni@NPCs are shown in Figs. 1d–h, S7 and S8, respectively. It can be clearly observed that the Mo_3_Ni_3_N polygonal nanosheets are regularly immobilized into the NPCs matrix, whose thicknesses are systematically measured to be about 3.4 nm, considerably smaller than those of Mo@NPCs (~ 4.2 nm). And such an ultra-thin nanosheets of Mo_3_Ni_3_N endow it with superior Li^+^ ions conductivity [25]. Besides, combined with the SAED results, the discernible lattice fringes with spacings of 2.21 Å, also recognized in the HRTEM image of Mo-Ni@NPCs, indexed to the Mo_3_Ni_3_N (221) crystal plane, which proves the existence of Mo_3_Ni_3_N phases for Mo-Ni@NPCs, as also confirmed by previous XRD results and EDS element mapping (Fig. 1b, g).

In the Raman spectra, there are two dominant peaks of Mo-Ni@NPCs, Mo@NPCs, NPCs and SP at 1334.35 cm^−1^ (D-band) and 1587.94 cm^−1^ (G-band) that can be recognized (Fig. S9), which are assigned to the graphite structural defects and tangential vibrations of the graphite phase. This demonstrates the formation of a microcrystalline graphite structure, thus endowing them with good electrical conductivity. It is noteworthy that *I*_D_/*I*_G_ value of Mo-Ni@NPCs is calculated to be about 0.9352, much lower than those of Mo@NPCs (1.0018), NPCs (1.0545) and SP (1.0469), meaning that its high degree of graphitization and structural orderliness, which can effectively accelerate electronic conductivity. This is of great importance as it will be dedicated to the enhancement of electron transfer ability in cathode for ASSLBs (Fig. S10). Besides, this result also illustrates that when compared with these counterparts, there exists the low content of graphite structural defects in Mo-Ni@NPCs, also identified by EPR results (Fig. S11), which can undermine the adsorption ability of oxygen-containing surface functional groups on them [26]. The N_2_ adsorption–desorption isotherms are examined to determine the specific surface area and pore size distribution for Mo-Ni@NPCs, Mo@NPC, NPCs and SP samples. Correspondingly, the BET specific surface area of Mo-Ni@NPCs is measured to be 20.39 m^2^ g^−1^, significantly lower than those of Mo@NPCs (48.60 m^2^ g^−1^), NPCs (53.59 m^2^ g^−1^) and SP (62.00 m^2^ g^−1^), respectively, which thus make it more difficult for oxygen-containing functional groups to survive in large numbers on the surface of Mo-Ni@NPCs (Fig. S12 and Table S1). These combined situations can effectively restrict the oxygen-containing functional groups-induced side reaction of SSEs inside in composite cathode, thus strengthening the performance of ASSLBs.

### Characterizations and Analyses of Side Reaction within Composite Cathodes

**Fig. 2:**
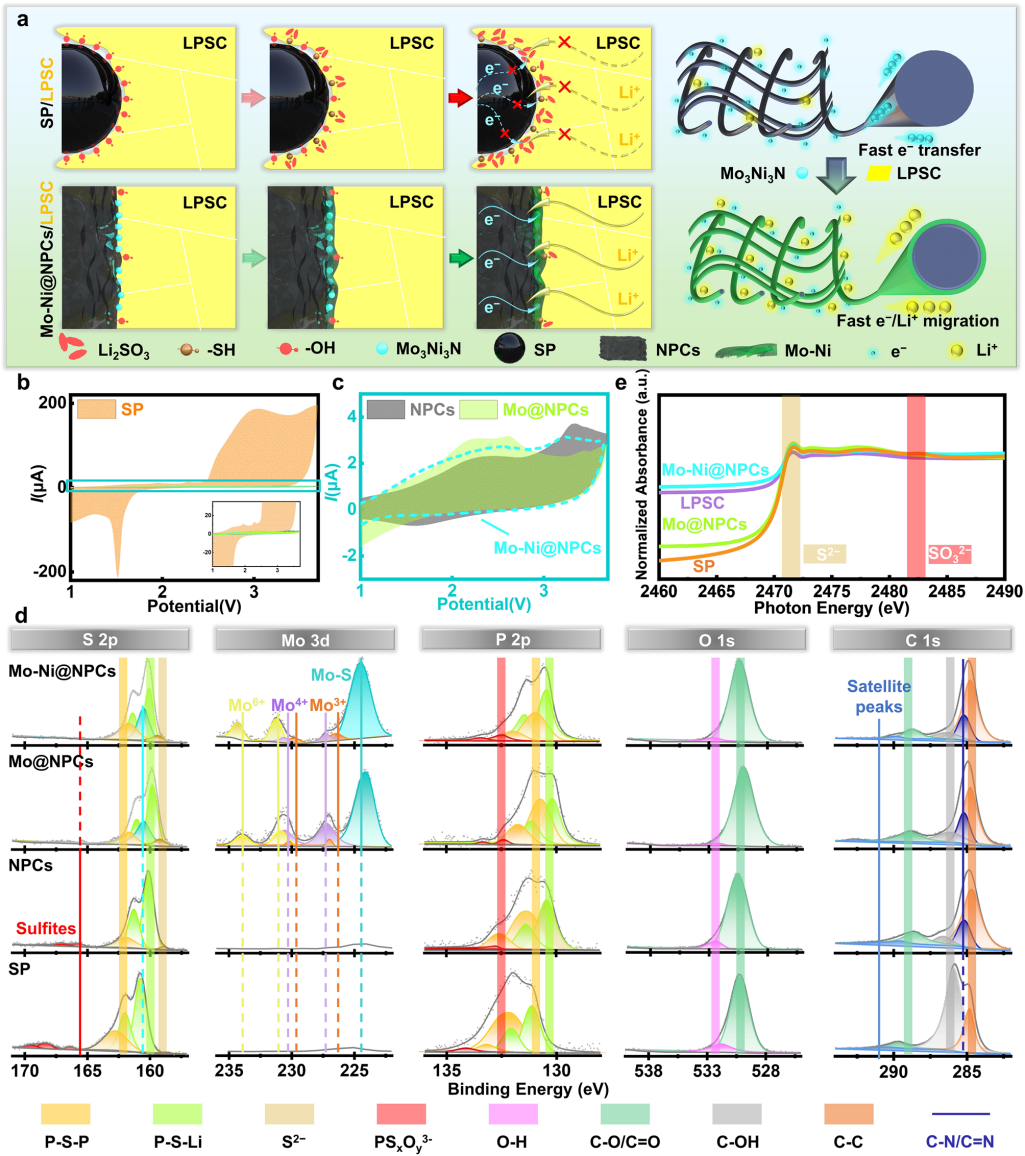
**a** Schematic diagram of interface reaction of the SSEs with Mo-Ni@NPCs and SP. **b-c** The 1st CV curve of SP/LPSC-based ASSLBs with non-AMs, NPCs/LPSC-based ASSLBs with non-AMs, Mo@NPCs/LPSC-based ASSLBs with non-AMs, and Mo-Ni@NPCs/LPSC-based ASSLBs with non-AMs. **d** XPS spectra of cathodes of SP/LPSC-based ASSLBs with non-AMs, NPCs/LPSC-based ASSLBs with non-AMs, Mo@NPCs/LPSC-based ASSLBs with non-AMs, and Mo-Ni@NPCs/LPSC-based ASSLBs with non-AMs after 100 cycles. **e** XANES spectra of S K-edge of LPSC, and the cathodes of SP/LPSC-based ASSLBs with non-AMs, Mo@NPCs/LPSC-based ASSLBs with non-AMs, Mo-Ni@NPCs/LPSC-based ASSLBs with non-AMs after 100 cycles (0.1C)

To directly investigate the occurrence situation of chemical/electrochemical side reactions at CCAs/SSEs interfaces, CV curves are obtained of ASSLBs with non-AMs for evaluation, which can effectively eliminate the interferences from other interfaces in the composite cathodes [27]. The CCAs assembled in composite cathodes, including Mo-Ni@NPCs, Mo@NPCs, NPCs and SP have distinctive oxidation/reduction peaks at 2.8/2.1 V (vs. Li^+^/Li) in the 1st CV curve, which are considered to be the key evidence for the occurrence of oxidation/reduction reaction of SSEs. Among them, the NPCs, Mo@NPC, and Mo-Ni@NPCs exhibit significantly lower oxidation–reduction peak currents than that of SP, indicating a giant inhibition of oxidation/reduction reaction of SSEs that due to the lower hydroxyl density on the surface. Surprisingly, the oxidation peaks at 3.4 V (vs. Li^+^/Li) are only found in cathodes with Mo@NPCs or Mo-Ni@NPCs, suggesting the formation of MoS_2_ on the at Mo@NPCs/SSEs and Mo-Ni@NPCs/SSEs interfaces (Figs. 2b, c and S13). Going further, the CV curves from 2nd to 5th cycles are obtained for ASSLBs with these samples in cathodes (Fig. S14). The test results demonstrate that when compared to the 1st CV cycle, all of them deliver a certain degree of attenuation of current peaks, but their relative intensity trends still remain unchanged, especially for Mo-Ni@NPCs, where there is still no appearance of oxidation–reduction peaks of SSEs, disclosing its stable anti-oxidation/reduction functionalities against LPSC for boosting performance of ASSLBs.

The XPS spectra of cathode composites materials after CV cycling are performed to observe variation in their corresponding chemical valence states, with results as shown in Figs. 2d and S15. From the high-resolution XPS S 2*p* spectra, it can be observed that the characteristic peaks at 160.08 and 161.43 eV correspond to the PS_4_^3−^ unit of argyrodites [7]. And the peaks appearing at 161.73 and 159.38 eV, can be assigned to P_2_S_x_ and S^2−^, respectively [7]. It can be also found that no sulfite species (165–170 eV) are detected in the cathode materials with Mo-Ni@NPCs and Mo@NPCs, whereas for NPCs and SP in cathodes, sulfite species are dramatically found in them. These sulfites are also identified by the XANES spectra of the S K-edge (Figs. 2e and S16), which is commonly considered a factor contributing to interface instability issues [28]. Interestingly, there is new peak appearing at 160.53 eV for the cathode with Mo-Ni@NPCs or Mo@NPCs after cycling, assigning to the formation of MoS_2_ phase in them, which is believed to be conducive to construction of Li^+^ ions migration channels, thus giving the improvement of Li^+^ ions transfer in these composite cathodes during cycling [28–31]. Furthermore, when compared to NPCs and SP in cathodes, the peak positions of the XPS S 2*p* spectra of cathodes with Mo-Ni@NPCs or Mo@NPCs shift negatively, which derives from the electrons transfer from Mo atoms to S atoms through Mo-S bonds due to the higher electronegativity of S atoms than Mo atoms. In the high-resolution XPS spectra of P 2*p*, the peaks at 130.38, 131.43, 130.98 and 131.98 eV are assigned to the PS_4_^3−^ -unit of argyrodites LPSC and P_2_S_x_. In addition, there is a new peak signal at 132.48 eV attributing to PS_x_O_y_^3−^ units that are indexed to the intermediate products, which are formed by the contact of LPSC with oxygen-containing species (-OH) on these surfaces [15]. Significantly, the sulfite peak at 165.73 eV is almost undetectable in the Mo-Ni@NPCs or Mo@NPCs-based cathodes, proclaiming their effective inhibition of side reactions with LPSC which means the intermediate products cannot undergo further side reactions to produce harmful sulfite species in this surface. In the detailed XPS spectra of Mo 3*d* for Mo-Ni@NPCs and Mo@NPCs (Fig. 2d), the peaks located at around 229.78 and 226.58 eV are associated with Mo 3*d*_5/2_ and Mo 3*d*_3/2_ of Mo^3+^ in MoN, respectively, while the peak of S 2*p* situated at about 159.38 eV, and peaks at about 229.78 and 226.58 eV are assigned to Mo 3*d*_5/2_ and Mo 3*d*_3/2_ of Mo^4+^ in MoS_2_, respectively, thus identifying the existence of MoS_2_-MoN and MoS_2_-Mo_3_Ni_3_N heterostructures in them, also confirmed by XRD and HRTEM results (Figs. 1b and S17–S18). Noticeably, it is clearly observed that Mo^3+^ peaks of Mo-Ni@NPCs and Mo@NPCs after cycling shift to a lower binding energy than those in the pristine counterparts (Fig. S5). This manifests that electrons are transferred from MoS_2_ to MoN and Mo_3_Ni_3_N, thus generating the built-in electric field for the MoS_2_-MoN and MoS_2_-Mo_3_Ni_3_N heterostructures, which may be advantageous to enhance the Li^+^ ions/electron transfer ability of the MoS_2_-MoN and MoS_2_-Mo_3_Ni_3_N heterostructures compared to their independent single-phases counterparts [24]. Besides, the peak at about 234.38 eV is a highly oxidized Mo 3*d*_3/2_ of Mo^6+^ state caused by surface oxidation of MoN and Mo3Ni3N phases [32]. The high-resolution N 1*s* XPS spectra can be deconvoluted into four prominent peaks. Aside from the three peaks at 397.03 eV for pyridinic-N species, 398.63 eV for pyrrolic-N species, and 402.38 eV for graphite-N species, respectively, a crucial peak at 395.08 eV could be examined for cathodes with Mo-Ni@NPCs and Mo@NPCs, which is indexed to the Ni/Mo–N bond due to existence of MoN and Mo_3_Ni_3_N phases in them. The high-resolution C 1*s* XPS spectra contain four main peaks centered at 284.8, 285.7, 286.9, 289.0, and 292.0 eV, representing C–C/C = C, C–N, C–OH, C = O/C–O and satellite peaks, respectively. In addition, the content of C–OH species in Mo-Ni@NPCs is calculated to be about 8.70%, considerably lower than those in Mo@NPCs (10.98%), NPCs (16.26%) and SP (12.79%) (Table S2). Moreover, the high-resolution O 1*s* XPS spectra contain two decomposed peaks centered at 530.23 and 531.93 eV, representing C–O/C = O and O–H, respectively. It should be noted that the –OH species in Mo-Ni@NPCs are calculated to be only 3.72%, which is considerably lower than those in Mo@NPCs (5.80%), NPCs (11.08%) and SP (14.43%) (Table S3). These results mean the Mo-Ni@NPCs attracts a low content of -OH groups, which is consistent with FTIR results (Fig. S19), thus emasculating the LPSC degradation reactions at cathode interfaces and hence stabilizing performance of ASSLBs (Fig. 2a) [15].

### Electronic States and Li^+^ Ions Diffusion Energy Barriers

**Fig. 3:**
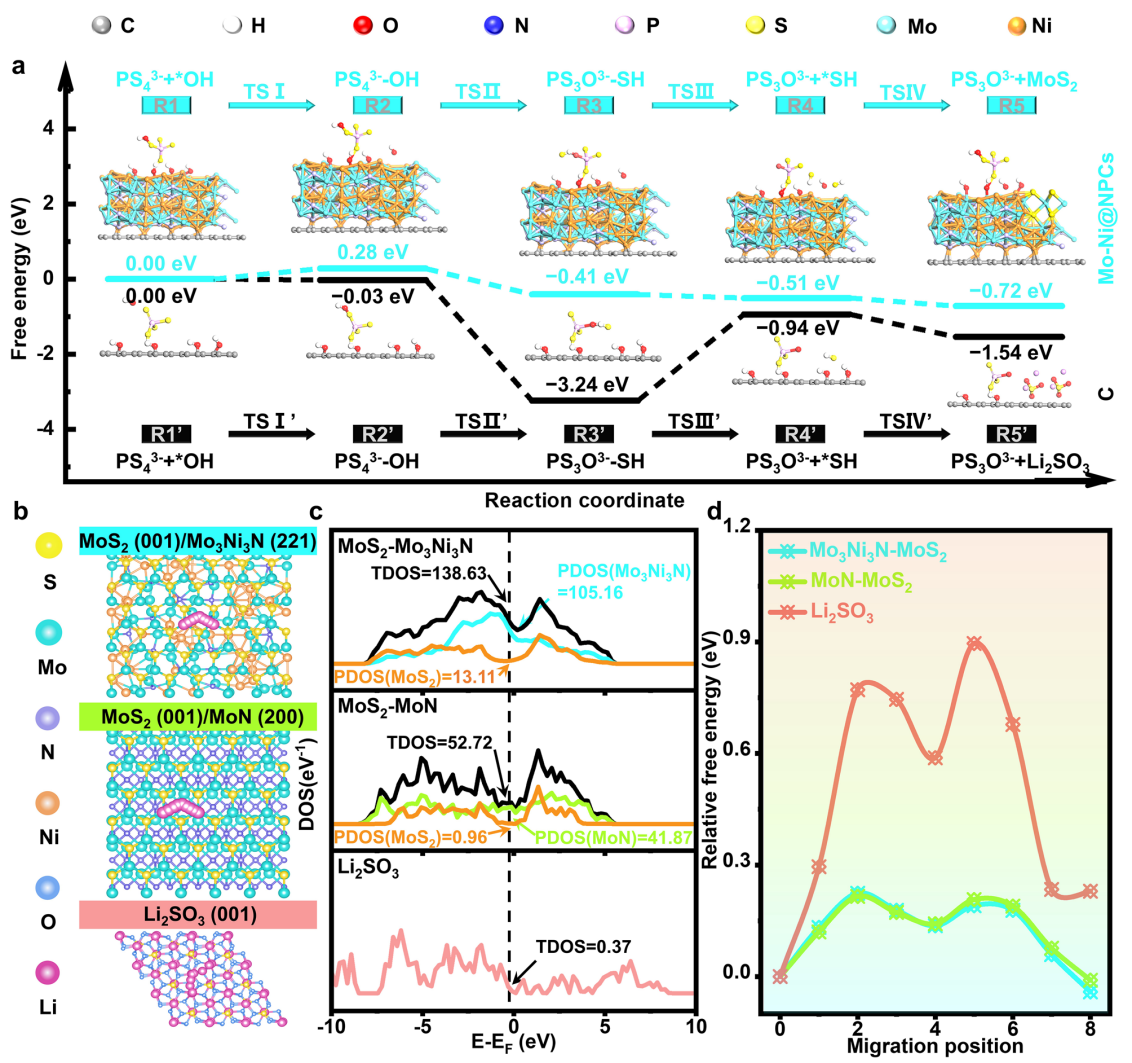
**a** Schematic diagram of side reactions and DFT-computed free energy diagrams of pathway on SP|LPSC interface and Mo-Ni@NPCs|LPSC interface. **b** Top view of migration pathways of Li^+^ ions on surfaces of MoS_2_-Mo_3_Ni_3_N heterostructure, MoS_2_-MoN heterostructure and Li_2_SO_3_. **c** DOS of MoS_2_-Mo_3_Ni_3_N heterostructure, MoS_2_-MoN heterostructure and Li_2_SO_3_. **d** Diffusion energy barriers of Li^+^ ions migration at different interfaces

To further unveil the kinetic characteristics of SSEs decomposition reaction at Mo-Ni@NPCs/SSEs interface and SP/SSEs interface in composite cathodes, the rational construction of Mo_3_Ni_3_N(221) and C(002) models with geometry optimizations are preferentially selected for exploring the LPSC degradation mechanism and inhibitory mechanisms, to be elucidated theoretically by DFT calculation (Fig. S20). Detailed descriptions are found in the Supporting Information. More concretely, the SSEs degradation mechanisms are proposed for C(002), including the main basic-steps involving PS_4_^3−^ + *OH, PS_4_^3−^–OH, PS_3_O^3−^–SH, PS_3_O^3−^ + *SH and PS_3_O^3−^ + Li_2_SO_3_ (Figs. 3a and S21). Corresponding mechanisms occurring with Mo_3_Ni_3_N(221) include the main species of PS_4_^3−^ + *OH, PS_4_^3−^–OH, PS_3_O^3−^-SH, PS_3_O^3−^ + *SH and PS_3_O^3−^ + MoS_2_. Whether generating MoS_2_ or Li_2_SO_3_ over them, the bond dissociation step of PS_3_O^3−^–SH to form PS_3_O^3−^ + *SH needs to overcome the largest energy barrier and can be perceived as the rate-determining step during the whole dissociation processes. It is noteworthy that the rate-determining step for producing MoS_2_ over Mo_3_Ni_3_N(221) is 1.06 eV, much lower than that for producing Li_2_SO_3_ over C(002) (3.07 eV), suggesting that the formation of MoS_2_ rather than Li_2_SO_3_ is more feasible over the Mo-Ni@NPCs. Besides, combining with XPS, FTIR, and EPR results, there are a small amount of -OH survived over the Mo-Ni@NPCs, which further lowers the generation of Li_2_SO_3_ through -OH groups-induced competitive degradation reaction of SSEs. This effectively leads to the formation of MoS_2_-Mo_3_Ni_3_N heterostructure while inhibiting the formation of harmful sulfite, thus building the high-efficient Li^+^ ions/electrons migration pathways in composite cathodes of ASSLBs. To further elaborate on these advantages of MoS_2_-Mo_3_Ni_3_N heterostructure in composite cathode, the basic structures models of MoS_2_, MoN, Mo_3_Ni_3_N, and Li_2_SO_3_ are architected by the Vienna Ab-Initio Simulation Package (VASP) method. Using this package, we construct the stable MoS_2_–MoN and MoS_2_–Mo_3_Ni_3_N due to their ultra-low lattice mismatch degrees of less than 2%, suggesting the rationality and effectiveness of these nano-structure models constructions (Figs. S22, S23 and Table S4). Accordingly, the electron density of states (DOS) corresponding to these optimized heterostructures are computed, as shown in Figs. 3c and S24, S25. It is scientifically observed that the DOS at the Fermi level of MoS_2_-MoN and MoS_2_-Mo_3_Ni_3_N heterostructures is significantly higher than that of Li_2_SO_3_, paving the way for excellent electronic conductivities, aided by synergistic effects between these constituent components [33]. The migration pathways and diffusion energy barriers of Li^+^ ions on the surfaces of the three samples are illustrated in Figs. 3b, d and S26, S27. The diffusion energy barriers of Li^+^ ions on the surfaces of MoS_2_-MoN and MoS_2_-Mo_3_Ni_3_N heterostructures are much lower than on Li_2_SO_3_, indicating the superior Li^+^ ions diffusion kinetics of these two heterostructures compared to Li_2_SO_3_. In order to further quantify electrode kinetics, a Galvanostatic intermittent titration technique (GITT) is conducted and diffusion coefficients within different batteries are simultaneously calculated, as shown in Fig. S28. During the initial charging stage, the Li^+^ ions diffusion coefficients of these materials are kept essentially identical [34]. Subsequently, as the voltage reaches a certain level, the Li^+^ ions diffusion coefficients of cathode materials containing Mo-Ni@NPCs and Mo@NPCs increase, manifesting that some new components formed within the cathode can enhance the Li^+^ ions mobility over them, attributed to the contribution of MoS_2_ formed over the Mo_3_Ni_3_N and MoN substrates [24]. These studies provide the theoretical and experimental foundation support for the excellent electron/Li^+^ ions dual-conductivity of Mo@NPCs and Mo-Ni@NPCs in composite cathodes for ASSLBs.

### In Situ Test and Visualization of Interface Side Reactions

**Fig. 4:**
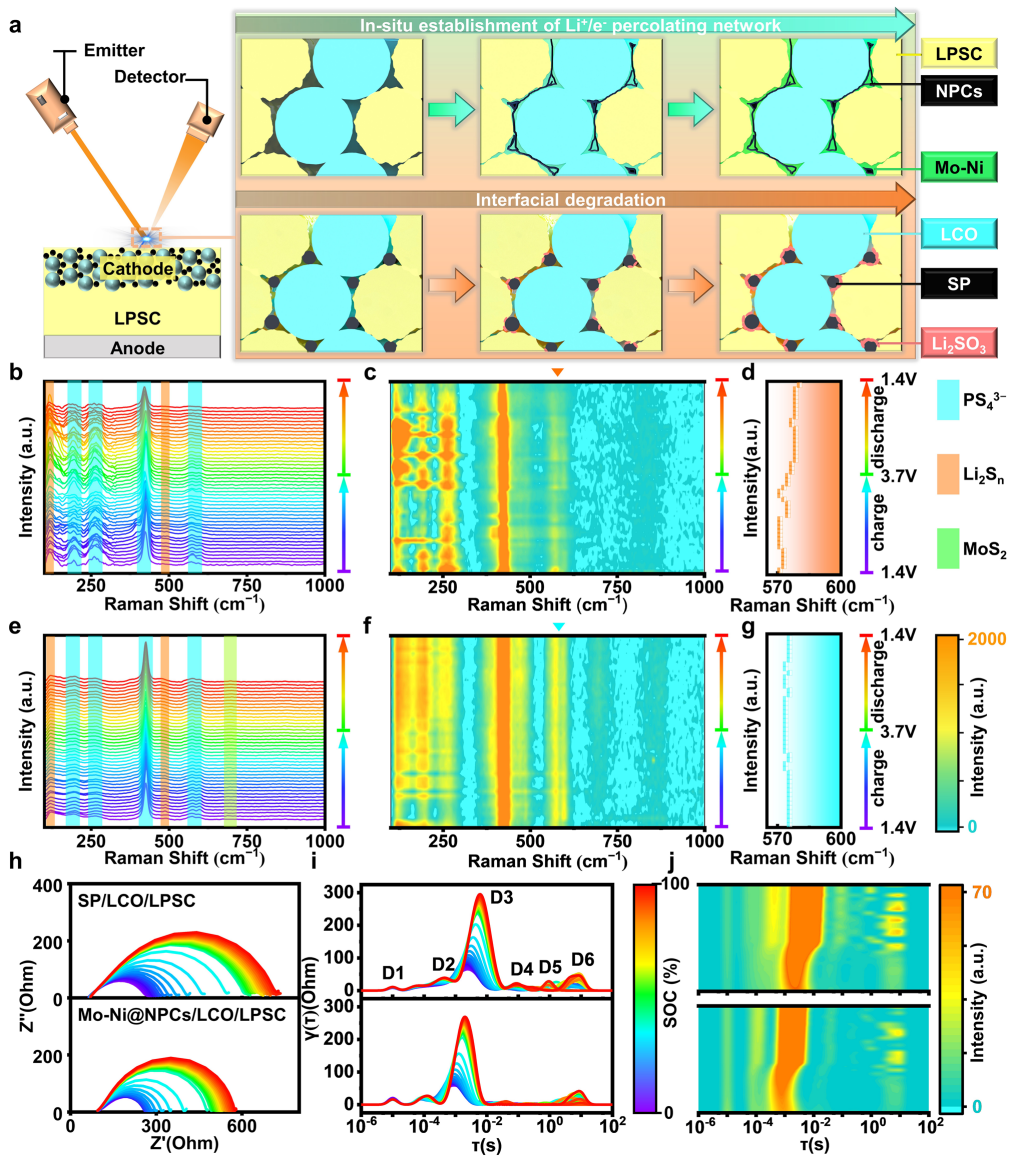
**a** Schematic diagram of *operando* Raman test. **b-g** *Operando* Raman spectra and the contour plots of cathodes of **b-d** SP/LCO/LPSC-based ASSLBs and **e–g** Mo-Ni@NPCs/LCO/LPSC-based ASSLBs. **h** In situ GEIS, **i** DRT analyses and** j** contour plots of DRT for SP/LCO/LPSC-based ASSLBs and Mo-Ni@NPCs/LCO/LPSC-based ASSLBs at 1st cycle during the charge process at 0.1C

The composition and structure evolution mechanism caused by the introduction of CCAs in composite cathode during the cycling is further explored by novel *operando* Raman spectroscopy (Fig. 4a), to dynamically monitor the distribution characteristics of LPSC degradation at cathodic interface accompanied with their influence on the interface stability. The *operando* Raman spectra of cathode materials containing Ni-Mo@NPCs, Mo@NPCs, NPCs and SP are depicted in Figs. 4b–g and S29, S30. Generally, the distinctive peaks at 200, 270, 425, and 570–600 cm^−1^ are corresponded to the PS_4_^3−^ unit of argyrodite LPSC. And the recognized peaks at 121 and 485 cm^−1^ are assigned to the Li_2_S_n_. Noticeably, the emergent peak at 708 cm^−1^ is considered to indicate the formation of MoS_2_ phase [35, 36]. Through contour plots, it is visually apparent that ASSLBs with Mo-Ni@NPCs and Mo@NPCs exhibit high stability, manifested by the unchangeable peak intensities of Li_2_S_n_ and PS_4_^3−^. Additionally, MoS_2_ is in situ generated at initial cycling stages and remains invariant in intensity throughout subsequent charge and discharge cycles. In contrast, ASSLBs with SP and NPCs exhibit distinct changes and even occur crossover phenomena for Li_2_S_n_ and PS_4_^3−^ peak intensities. These results visually announce that the introduction of Mo-Ni@NPCs and Mo@NPCs into composite cathodes can furnish the remarkable cathode interface stability, however, an opposite effect is observed for SP and NPCs. Mo-Ni@NPCs and Mo@NPCs effectively inhibit LPSC degradation reaction happened at the interfaces, which thus prevent the formation of harmful Li_2_SO_3_ from filling the cathode interfaces to hinder the transfer of electron/Li^+^ ions. In addition, in situ Raman spectra after different cycles are used to further demonstrate Mo@NPCs and Mo-Ni@NPCs Can effectively improve interface stability (Fig. S31).Thus, Mo-Ni@NPCs and Mo@NPCs contribute to the improvement of ASSLBs performance.

The in situ galvanostatic electrochemical impedance spectra (GEIS) are executed to uncover the cathode evolution of ASSLBs during cycling, and the intercepts of the GEIS with the X-axis at high frequency region represent the ohmic resistances of ASSLBs, while the semicircles at med/low-frequency region stand for the interfacial resistances for Li^+^ ions transfer. As seen in Figs. 4h and S32–S34, it is demonstrated that all the semicircles become enlarged during progress through sequential states of charge (SOC), which are mainly triggered by the corresponding cathode interface evolution [37]. The ASSLBs with Mo-Ni@NPCs afford the smallest change in interfacial resistances during charging processes at 0.1C, 0.2C and even 0.5C, expanding from approximately 282.86 to 579.23 Ω, 442.59 to 1168.41 Ω, 630.85 to 1194.86 Ω, which are all far less than the changes in Mo@NPCs, NPCs, and SP assembled in ASSLBs, suggesting there is stable and fast interfacial diffusion kinetics in the Mo-Ni@NPCs/LCO/LPSC cathode composites during the electrochemical processes.

To accurately distinguish the specific evolution of the cathodes composites interfaces, the distribution of relaxation time (DRT) is employed. This can recognize the time domain-based spectra with the help of a mathematical transformation of the frequency domain-based Nyquist plots [38]. The accomplished relaxation-based function γ(τ) can convey the specialized electrochemical processes by the generation of characteristic peaks at the specific relaxation time. Accordingly, the DRT technology can decouple the resistance evolution in cathode composites during the charging/discharging processes. To be mentioned, the cathode composite interfaces assume better structural stability during charge processes than during discharge processes. As seen in Figs. S32–S34, there is more drastic variation in GEIS and DRT results for discharge processes compared to charging processes, demonstrating the sophisticated changes in the multi-scale Li^+^ ions transport kinetic at the interfaces (such as cathodic interface, anodic interface, grain boundary of LPSC), and thus the charging process is more desirable for the researching the influence of CCAs on cathode interface changes and stabilities.

As shown in Fig. 4h and S32–S34, the six dominant peaks are observed during charge process at 0.1C, 0.2C and 0.5C (labeled as D1, D2, D3, D4, D5, and D6). The D1 peak at high frequency of around 105 Hz is attributed to the ground boundaries interfaces of LPSC [39]. The D2 expresses the solid electrolyte interphase (SEI) at anodic interfaces and the D3 peak represents the cathodic interface resistance. The D4 and D5 peaks are relevant to the charge transfer (CT) at Li-In/LPSC and CCAs/LCO/LPSC interfaces. And D6 peak with the lowest frequency (> 1 Hz) are derived from the solid-state diffusion within LCO composite cathodes [40]. The D1 peak is detected in SP/LCO/LPSC-based ASSLBs and Mo-Ni@NPCs/LCO/LPSC-based ASSLBs, whose peaks intensities remain extremely stable and are almost independent of SOC, indicating that it can be attributed to the intrinsic stability of LPSC component, which has been demonstrated to exist steady in ASSLBs, thus also verifying the accuracy and precision of DRT technique [39, 40]. The D2 and D4 peaks referring to anodic interfaces (SEI and CT) hold the desirable stability during the whole charge process, owing to the great enhancement by the introduced LPSC electrolytes. which eliminate the cross interference between the anodic side and the cathodic side, and the discrepancy of cycling performance of ASSLBs can only be ascribed to cathode interface compatibility differences. The D3 associates with Li^+^ ions transfer ability at cathode interface, and the evident variations happen during charging processes and similar variation trends are recognized for them. Although the D3 peak eventually stabilizes for them, the intensity growth of D3 peak in Mo-Ni@NPCs/LCO/LPSC-based ASSLBs is much lower than in SP/LCO/LPSC-based ASSLBs, manifesting its further restricted LPSC degradation which produces insulating sulfates at the cathode interfaces. As for D5 peak relating to electron transfer at cathode interface, it is noting that when compared with counterparts, almost no peak intensity for this peak is observed for Mo-Ni@NPCs/LCO/LPSC-based ASSLBs, implying its superior electron conductivity in composite cathode. Overall, the building of distinguished Li^+^ ions/electrons transfer pathways are materialized by the introduction of Mo-Ni@NPCs into composite cathode, which has been enlightened by both GEIS and DRT methods described herein.

### Performance Verification and Analysis of ASSLBs

**Fig. 5:**
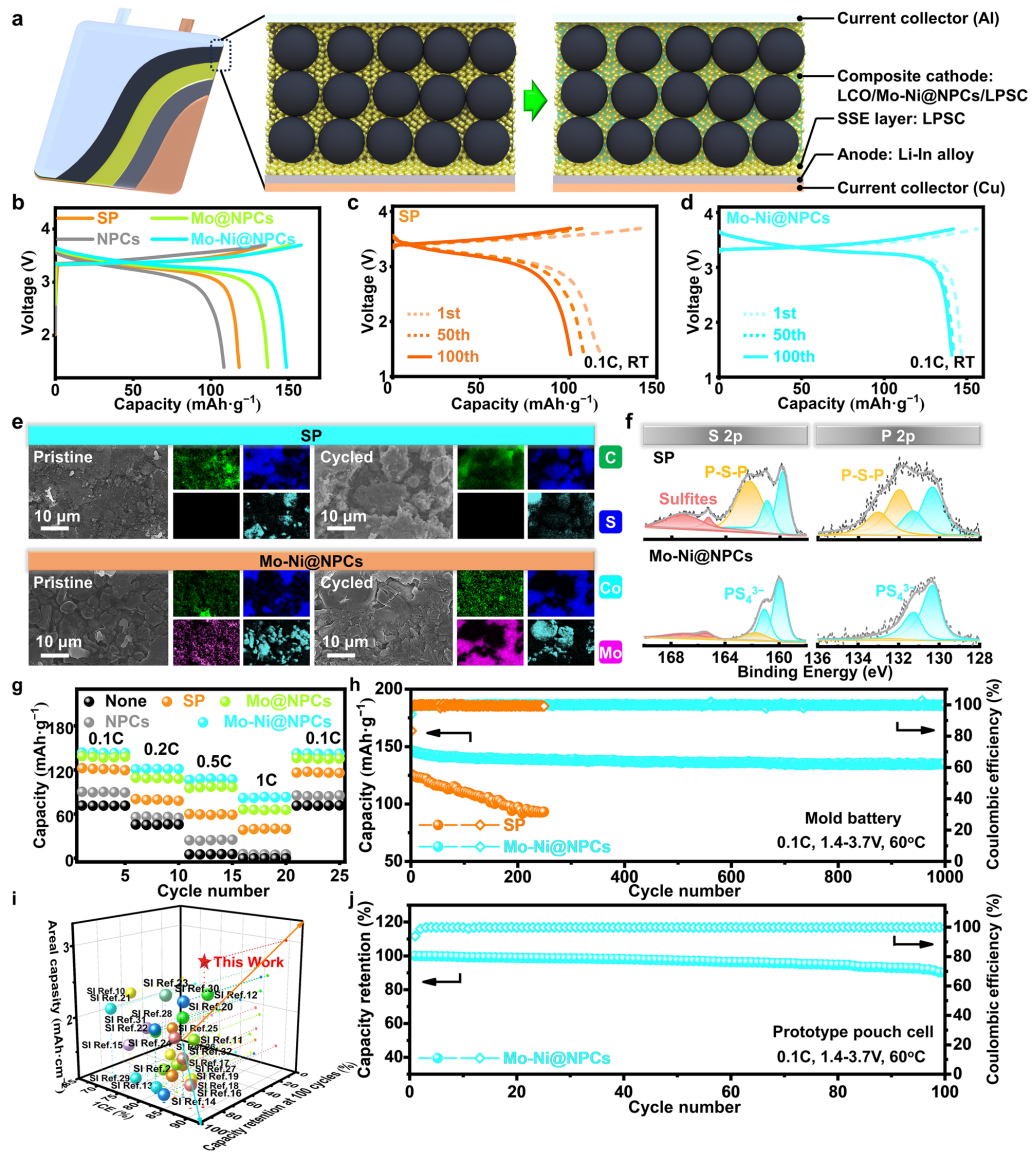
**a** Schematic diagram of structure of the prototype pouch cell. **b** Effects of 2% CCAs on the 1st cycle charge/discharge performance of ASSLBs (0.1C). **c** Charge/discharge performance of ASSLBs with 2% SP at different cycles (0.1C).** d** Charge/discharge performance of ASSLBs with 2% Mo-Ni@NPCs at different cycles (0.1C). **e** SEM images and EDS elemental mapping of cathodes of SP/LCO/LPSC-based ASSLBs and Mo-Ni@NPCs/LCO/LPSC-based ASSLBs before and after 100 cycles. **f** S 2*p* and P 2*p* of XPS spectra of SP/LCO/LPSC-based ASSLBs and Mo-Ni@NPCs/LCO/LPSC-based ASSLBs after 100 cycles.** g** Rate performance of ASSLBs. **h** Long cycle stability of SP/LCO/LPSC-based ASSLBs and Mo-Ni@NPCs/LCO/LPSC-based ASSLBs at 60 °C (0.1C). **i** The performance in reported literature compared with our work. **j** Cycling performance and coulombic efficiency of Mo-Ni@NPCs/LCO/LPSC-based prototype pouch cell (1.8 mAh) are plotted against the cycle numbers. A constant current (CC) model with the charge/discharge rate of 0.1C/0.1C is applied (voltage windows, 1.4–3.7 V vs. Li^+^/(In-InLi) at 60 °C). The area capacity loading of the LCO cathodes is 0.15 mAh cm^−2^ (0.1C = 0.015 mA cm^−2^)

The influences of Mo-Ni@NPCs, Mo@NPCs, NPCs and SP assembled in composite cathodes on the performance of ASSLBs are assessed by the battery tester. The 1st charge–discharge capacities and Coulombic efficiencies of ASSLBs with different concentration of these CCAs are illustrated in Fig. S35 and Table S5. Notably, Mo-Ni@NPCs/LCO/LPSC-based ASSLBs exhibits the highest discharge capacities of about 136.95 mAh g^−1^ and the highest Coulombic efficiency of about 90.36% at 0.1C, however, SP/LCO/LPSC-based ASSLBs only provides a capacity of 111.58 mAh g^−1^ and Coulombic efficiency of around 81.84% at 0.1C. Additionally, in nearly all reports of this study, the introduction of CCAs results in an additional slope before reaching the LCO plateau voltage during the 1st charge, assigned to the oxidation of SSEs into other products during the charging process [16, 41, 42]. Importantly, almost all the samples assembled in cathodes attain the highest capacity and Coulombic efficiency at a content of 2%. This is mainly because that the moderate addition of CCAs ensures high electron conductivity, and also lowers the generation of sulfites through SSEs degradation at the cathode interfaces, thus improving the overall performance of battery. On this grounds, further comparisons of the charge–discharge performances of ASSLBs with different CCAs are performed at the 2% content addition level, as shown in Fig. 5b, revealing that Mo-Ni@NPCs delivers the charge/discharge capacities greater than other counterparts, owing to its comprehensive effects of high electron conductivity, desirable Li^+^ ions migration rate and especially restriction of the oxidation decomposition of SSEs, thus strengthening the cathode interface stability. Furthermore, long-term cycling stability of batteries is tested at 0.1C under room temperature (RT) conditions (Figs. 5c, d and S36–S38). Encouragingly, Mo-Ni@NPCs/LCO/LPSC-based ASSLBs still presents excellent long-term cycling stability, with an ultra-high capacity retention rate of 93.26% after 100 cycles at RT, significantly higher than SP (85.52%), NPCs (79.81%), and Mo@NPCs (91.91%). It is important to note that while ASSLBs without CCA exhibit excellent stability, the lack of an electronic conductive network in the composite cathode results in relatively low capacity (~ 71 mAh g^−1^) [15].

The situation of composite cathode interfaces before and after cycling can be directly visualized and analyzed by SEM characterization and corresponding EDS elemental mapping, as shown in Figs. 5e and S39–S46. These demonstrate that the cathode interfaces of Mo@NPCs/LCO/LPSC-based ASSLBs and Mo-Ni@NPCs/LCO/LPSC-based ASSLBs disclose the enrichment of Mo and S elements, rather than S and O elements. They differ from the results of the cathode interface of SP/LCO/LPSC-based ASSLBs and NPCs/LCO/LPSC-based ASSLBs. In addition, the cracks sites appear in the cathode interfaces of SP/LCO/LPSC-based ASSLBs and NPCs/LCO/LPSC-based ASSLBs, owing to the generation of a large amounts of sulfites which cause the projection of internal stress, which is not conducive to cycling stability of ASSLBs. By comparison, the composite cathode interfaces containing Mo@NPCs and Mo-Ni@NPCs preserve the tight contact with each other after cycling, due to almost no formation of sulfites among them, which is also corroborated by XPS spectra of ASSLBs before and after cycling (Figs. 5f and S47). Rate test is commonly used to evaluate battery stability (Fig. 5g), and it is evident that Mo-Ni@NPCs/LCO/LPSC-based ASSLBs maintains the highest capacity and stability, far superior to other counterparts assembled ASSLBs. Further long-term cycling stability testing of different batteries is conducted at a fixed charge/discharge rate at 0.1C/0.1C under a high temperature of 60 °C (Figs. 5h and S48, S49), revealing the most outstanding cycling stability of Mo-Ni@NPCs/LCO/LPSC-based ASSLBs, with a capacity retention rate of 90.62% after 1000 cycles at this high temperature, thus demonstrating the excellent performance. These evaluations collectively illustrate that Mo-Ni@NPCs not only improves cathode stability but also enhances battery capacity and capacity retention. To further evaluate the widespread application prospects of Mo-Ni@NPCs, the 1st charge–discharge curves of Mo-Ni@NPCs/NCM/LPSC-based ASSLBs and SP/NCM/LPSC-based ASSLBs are presented in Figs. S50 and S51. The Mo-Ni@NPCs maintains superior discharge capacity and Coulombic efficiency compared to SP in NCM-based cathodes. Additionally, the cathodes containing Mo-Ni@NPCs consistently exhibit higher slopes before charging to a plateau voltage, suggesting its far-reaching commercial potential. At present, one of the requirements for the industrialization of ASSLBs is to achieve high capacities and excellent stabilities of composite cathodes materials under conditions of large areal capacities. The long-term cycling performances of ASSLBs with different areal capacity conditions using SP and Mo-Ni@NPCs are exhibited in Figs. S52 and S53. Interestingly, even under the condition of higher areal capacity of 3.00 mAh cm^−2^, the 1st cycle discharge specific capacity of ASSLBs with added Mo-Ni@NPCs at 0.1C can reach 127.55 mAh g^−1^, with a 1st Coulombic efficiency of 89.28%, and the capacity retention rate after 100 cycles exceeds 82.59%, outperforming the representative composite cathodes assembled ASSLBs as recently reported (Fig. 5i and Table S6). The charge/discharge curves for a capacity of 1.8 mAh in the Mo-Ni@NPCs/LCO/LPSC-based prototype pouch cell and the SP/LCO/LPSC-based prototype pouch cell at 0.1C/0.1C charge/discharge rate reveal first-cycle discharge capacities of 128.25 and 77.58 mAh g^−1^, corresponding to 90.84% and 85.48% of the discharge capacities of model batteries, respectively (Figs. 5a, j and S54). Notably, the cycling performance and corresponding Coulombic efficiencies of the prototype pouch cells demonstrate that the Mo-Ni@NPCs/LCO/LPSC-based prototype pouch cell outperforms the SP/LCO/LPSC-based prototype pouch cell. Specifically, the Mo-Ni@NPCs/LCO/LPSC-based prototype pouch cell retains 96.34% and 90.18% of discharge capacity after 60 and 100 cycles at 0.1C, respectively, which surpasses the performance of the SP/LCO/LPSC-based prototype pouch cell. Furthermore, the first-cycle Coulombic efficiency of the Mo-Ni@NPCs/LCO/LPSC-based prototype pouch cell at 0.1C is 93.89%, which is much higher than that (83.75%) observed for the SP/LCO/LPSC-based prototype pouch cell. These improvements are attributed to the enhanced stabilization of the composite cathode interface achieved through the “conversion-protection” mechanism introduced by the Mo-Ni@NPCs, thus further demonstrating its commercial feasibility and prospects.

## Conclusions

In summary, this research successfully fabricates the Mo-Ni@NPCs with the low densities of graphic defects sites and low contents of oxygen-containing groups, by using a sequential approach involving reverse emulsion polymerization, impregnation, and carbonization treatment, aiming at mitigating the SSEs degradation at composite cathode interface. It is worth noting that the Mo-Ni@NPCs in ASSLBs undergoes in situ generation of MoS_2_ at the cathode interface instead of generating sulfites during battery cycling, primarily due to the preferential oxidation reaction of Mo_3_Ni_3_N rather than LPSC. This transformation not only enhances the stability of the cathode but also facilitates the in situ generation of ultra-stable MoS_2_-Mo_3_Ni_3_N heterostructures with excellent electronic conductivity and Li^+^ ions migration rates. Performance evaluations indicate that at 0.1C, ASSLBs with Mo-Ni@NPCs exhibits an excellent 1st discharge specific capacity of 148.61 mAh g^−1^, with high Coulombic efficiency of 94.01% and a high capacity retention rate of 90.62% after 1000 cycles, outperforming the ASSLBs with SP. At high areal capacity conditions of 3.00 mAh cm^−2^, this battery still reaches a high discharge capacity of 127.55 mAh g^−1^ at 0.1C, a satisfactory Coulombic efficiency of 89.28%, extraordinary capacity retention rate of 82.59%, and outstanding long-term cycling stability of 100 cycles. This work addresses the challenges of composite cathode interface instability through the “conversion-protection” approach, thereby enhancing the electrochemical performance of batteries and providing new insights for researchers to address the knotty issues of composite cathode instability. Additionally, this approach promotes the commercialization of high-capacity ASSLBs, warranting further exploration by researchers in this field.

## Supplementary Information

Below is the link to the electronic supplementary material.

Supplementary file1 (DOCX 5534 kb)
