# Supplementary material for: Construction of Multifunctional Conductive Carbon-Based Cathode Additives for Boosting Li6PS5Cl-Based All-Solid-State Lithium Batteries
Source: Nanomicro Lett. 2025 Feb 11;17:140. doi: 10.1007/s40820-025-01667-7 (PMC11813843; doi:10.1007/s40820-025-01667-7)
Supplement: Supplementary file 1 — Supplementary file1 (DOCX 5534 kb) [file 40820_2025_1667_MOESM1_ESM.docx]

Supporting Information of

**Construction of Multifunctional Conductive Carbon-Based Cathode Additives for Boosting Li_6_PS_5_Cl-Based All-Solid-State Lithium Batteries**

Xin Gao^1,†^, Ya Chen^1,†^, Zheng Zhen^1,†^, Lifeng Cui^1, 2,^ *, Ling Huang^5^, Xiao Chen^1^, Jiayi Chen^1^, Xiaodong Chen^3,^*, Duu-Jong Lee^3,^*, Guoxiu Wang^4,^*

^1^ College of Smart Energy, Shanghai Jiao Tong University, Shanghai 200240, P. R. China

^2^ Smart Materials for Architecture Research Lab, Innovation Center of Yangtze River Delta, Zhejiang University, Jiashan 314100, P. R. China

^3^ Department of Mechanical Engineering, City University of Hong Kong, Tat Chee Avenue, Kowloon 999077, P. R. China

^4^ Centre for Clean Energy Technology, School of Mathematical and Physical Science, Faculty of Science, University of Technology Sydney, Sydney, 2007, Australia

^5^ School of Physical Science and Technology, Shanghai Tech University, Shanghai 201210, P. R. China

† Xin Gao, Ya Chen, and Zheng Zhen contributed equally to this work.

*Corresponding authors. E-mail: [lifeng.cui@gmail.com](mailto:lifeng.cui@gmail.com) (Lifeng Cui); [xche24@cityu.edu.hk](mailto:xche24@cityu.edu.hk) (Xiaodong Chen); [tuclee@cityu.edu.hk](mailto:tuclee@cityu.edu.hk) (Duu-Jong Lee); [Guoxiu.Wang@uts.edu.au](mailto:Guoxiu.Wang@uts.edu.au) (Guoxiu Wang)

**S1 Materials and Methods**

**S1.1 Materials Characterizations**

***S1.1.1 Fourier Transform Infrared Spectroscopy (FTIR)***

The functional groups on the surface of the simples are characterized by FTIR (Nicolet iS10) with the wavenumber ranging from 4000 to 500 cm-1. FTIR spectra are baseline calibrated and smoothed using Omnic 8.0 software.

***S1.1.2 N_2_ Adsorption/Desorption Measurements***

The specific surface area and N_2_ adsorption/desorption isothermal curves of samples are characterized by surface area and porosity analyzer (Micromeritics TriStar II 3flex).

***S1.1.3 Characterization of Material Morphology***

The morphologies of the samples are characterized using scanning electron microscopy (SEM, Zeiss Gemini 300), Atomic Force Microscope (AFM, Bruker Dimension ICON), HAADF-STEM (Thermo Themis Z) and high-resolution transmission electron microscopy (HRTEM, JEOL JEM 2100F), with further elemental distribution analysis conducted via energy-dispersive spectroscopy (EDS).

***S1.1.4 Raman Spectroscopy***

Raman (Thermo Fischer DXR) spectra are employed to determine the structure of the samples with the wavenumber ranging from 1000 to 2000 cm^-1^.

***S1.1.5 Thermogravimetric (TG)***

TG (Mettler Toledo TGA2) curves are used to test the temperature resistance and carbonization temperature of nano-sponge. To quantify the thermal decomposition temperatures of the samples, the temperature is increased by 10 °C/min from room temperature (25 ℃) to 1000 °C, through a ramp function.

***S1.1.6 Electron Paramagnetic Resonance (EPR)***

EPR (Bruker EMXplus) curves are utilized to characterize the density of carbon defects in the materials with the g-value is 2.0023.

***S1.1.7 Electronic Conductivity***

The electronic conductivity of the as-synthesized materials (Jingke ST2722-SZ powder resistivity tester) is tested under a pressure of 4 MPa using the four-probe method.

***S1.1.8 X-ray Powder Diffraction (XRD)***

XRD measurements are carried out over a 2θ range of 10-70° (X'Pert PRO MPD) with a Cu Kα line as the radiation source.

***S1.1.9 X-ray Photoelectron Spectroscopy (XPS)***

XPS (Thermo ESCALAB 250XI) spectra are used to evaluate the molecular structure and atomic valence states in both the carbon materials and solid-state battery cathodes. Monochromatic Al Kα radiation is used for analysis (3000 eV). The power of the X-ray source is 50 W, and the beam voltage is 15 kV. The beam diameter is 900 μm. Depth profiling is performed using Ar^+^ ions (soft sputtering with 0.5 kV) to clean the surface and to avoid misinterpretations due to reactions that occur only on the surface. The rasterized area is (6×6) mm^2^. The samples are transferred to the analysis chamber in an argon-filled transfer bin in order to avoid air exposure.

***S1.1.10 X-ray Absorption Near-edge Structure (XANES)***

S K-edge XANES spectra are measured at the BL08U1-A beamline at the Shanghai Synchrotron Radiation Facility, and the data are used to further characterize changes in molecular structure and atomic valence states of the cathode materials after cycling.

***S1.1.11 Operando Raman***

The *operando* Raman spectra are test by Renishaw inVia Qontor instrument with the wavenumber range is from 100 to 800 cm^-1^. And the batteries for the operando Raman characterizations are sealed in the test mold, which cycle at 0.1C (1 C=160 mA·g^-1^).

**S1.2 Electrochemical Measurements**

***S1.2.1 Cyclic Voltammetry (CV)***

The CV curves within the voltage range of 1.0-3.7V (scan rate: 0.01 mV/s) for the ASSLBs without active materials.

***S1.2.2 In-situ Galvanostatic Electrochemical Impedance Spectroscopy (in-situ GEIS)***

The *in-situ* GEIS curves (the frequency ranges from 0.1 to 10^6^ Hz) during charge-discharge processes at a current density of 78.4 μA (0.1C) for the SP/LCO/LPSC-based ASSLBs, NPCs/LCO/LPSC-based ASSLBs, Mo@NPCs/LCO/LPSC-based ASSLBs, Mo-Ni@NPCs/LCO/LPSC-based ASSLBs, which are measured using a Bio-Logic electrochemical workstation to uncover the cathode evolution of ASSLBs during cycling.

***S1.2.3 Galvanostatic Electrochemical Impedance Spectroscopy (GEIS)***

The ASSLBs are subjected to EIS testing using the same electrochemical workstation before and after 100 cycles at 0.1C and room temperature, covering a frequency range from 0.1 to 10^6^ Hz.

***S1.2.4 Galvanostatic Intermittent Titration Technique (GITT)***

The GITT curves during charge-discharge processes at a current density of 78.4 μA (0.1C) for the SP/LCO/LPSC-based ASSLBs, NPCs/LCO/LPSC-based ASSLBs, Mo@NPCs/LCO/LPSC-based ASSLBs, Mo-Ni@NPCs/LCO/LPSC-based ASSLBs, which are measured using a Bio-Logic electrochemical workstation to evaluate their Li^+^ ions transport efficiency.

***S1.2.5 Cycling Performance***

Furthermore, the ASSLBs are tested for charge-discharge cycling curves within the voltage range of 1.4V to 3.7V using a Neware battery tester to evaluate charge-discharge curves under various current densities, areal capacities, and temperatures (room temperature and 60°C), thereby comprehensively assessing their capacity, coulomb efficiency, and stability.

**S1.3 Assembly and electrochemical measurements of ASSLBs**

***S1.3.1 ASSLBs without Active Materials***

The carbon material and LPSC are mixed in a weight ratio of 1:5 in an agate mortar and pestle for 20 minutes to obtain the cathode without AMs. The Li/In alloy (with a mass ratio of Li to In of 1:30) served as the counter electrode. The ASSLB without AMs is employed to investigate the production of chemical/electrochemical by-products between the carbon material and SSEs. The assembly process is as follows: 100 mg of LPSC is added to a mold with a diameter of 10 mm and pressed at 10 MPa for 5 minutes to form the SSE. Subsequently, 7 mg of the cathode without AMs is evenly spread on one side of the SSE and pressed at 300 MPa for 10 minutes to ensure intimate contact with the SSE. Finally, 186 mg of Li/In negative electrode is uniformly spread on the other side of the SSE, and the assembly of the ASSLBs is completed by pressing at 30 MPa for 1 minute. These ASSLBs are named SP/LCO/LPSC-based ASSLBs with no-AMs, NPCs/LCO/LPSC-based ASSLBs with no-AMs, Mo@NPCs/LCO/LPSC-based ASSLBs with no-AMs, Mo-Ni@NPCs/LCO/LPSC-based ASSLBs with no-AMs, respectively.

***S1.3.2 ASSLBs with Active Materials***

The CCAs, LPSC, and LCO are mixed in an agate mortar and pestle in a weight ratio of 2:28:70 for 20 minutes to obtain the composite cathode. The Li/In alloy (with a mass ratio of Li to In of 1:30) served as the counter electrode [S1]. The assembly steps of the ASSLBs are identical to those of the ASSLBs without active materials. These ASSLBs are named SP/LCO/LPSC-based ASSLBs, NPCs/LCO/LPSC-based ASSLBs, Mo@NPCs/LCO/LPSC-based ASSLBs, Mo-Ni@NPCs/LCO/LPSC-based ASSLBs, respectively.

***S1.3.3 Preparation of Pouch Cell***

The composite cathode materials of pouch cell are prepared by mixed with LCO, LPSC and conductive carbon at the mass ratio of 70:25:5 for 20 min in a mortar by hand. To fabricate a dry film of the cathode, the above-prepared powder electrode is mixed with 1.5 wt% polytetrafluoroethylene (PTFE) in a heated mortar. After 3 min of mixing and shearing, a single flake is formed [S2].

The 45 mm × 55 mm pouch cell with an area capacity of 1.50 mAh·cm^-2^ is produced [S3]. Electrodes and SSE sheets are cut by a punching machine. The dimensions of the anode, cathode, and solid electrolyte sheet are 30 mm × 40 mm, 30 mm × 40 mm, and 35 mm × 45 mm, respectively. All electrodes and electrolyte sheets are stacked and packaged in a laminated bag. After the vacuum is applied to the laminate bag and sealed, the battery is pressurized for 10 min by a Warm Isostatic Press (WIP400, Sichuan Lineng Ultra High Voltage Equipment Co., Ltd) at a pressure of 300 MPa. Then, the battery is taken out of the laminate bag and the Al and Ni terminals of the cathode and anode, respectively, are welded using an ultrasonic welder. The battery is placed in a laminated bag and vacuum-sealed again. Most of the assembly process is carried out in the argon atmosphere.

**S1.4 DFT Calculation Details**

***S1.4.1 Diffusion Energy Barriers of Li^+^ Ions Migration at Different Interfaces***

This study employs density functional theory (DFT) to comprehensively analyze the mechanism of Mo@NPCs/LCO/LPSC-based ASSLBs and Mo-Ni@NPCs/LCO/LPSC-based ASSLBs. In the DFT calculations, the projector-augmented wave (PAW) method with a cutoff energy of 400 eV [S4] and the Perdew-Burke-Ernzerhof (PBE) functional [S5] are utilized. The DFT-D3 method [S6] is employed to correct for the van der Waals interactions. Heterostructure models of MoS_2_-Mo_3_Ni_3_N are established by cutting one layer of Mo_3_Ni_3_N (221) surface and one layer of MoS_2_ (001) surface with a 15 Å vacuum layer. Similarly, heterostructure models of MoS_2_-MoN are established by cutting two layers of MoN (200) surface and one layer of MoS_2_ (001) surface with a 15 Å vacuum layer. Additionally, a ribbon model of Li_2_SO_3_ is established by cutting two layers of Li_2_SO_3_ (001) crystal surface with a 15 Å vacuum layer, where half of the bottom atomic layer is fixed to simulate the bulk phase. All models are optimized with energy and force convergence criteria of 10^-5^ eV and 0.02 eV/Å, respectively. Brillouin zone integration is performed using the Monkhorst-Pack scheme. Furthermore, to obtain the energy barriers of Li^+^ ions migration on the surface of the bulk phase, the climbing-image nudged elastic band (CI-NEB) method is employed with a path threshold of 0.05 eV/Å.

***S1.4.2 DFT-computed Free Energy Diagrams of Pathway at Different Interfaces***

In addition, the free energy is performed using a castep module of Material Studio 2020. The generalized gradient approximation (GGA) method with Perdew-Burke-Ernzerhof (PBE) function is employed to describe the interactions between the valence electrons and the ionic core. The energy cut-off for the plane-wave basis set is 450 eV. The threshold values of the convergence criteria are specified as follows: 0.001 Å for maximum displacement, 0.03 eV Å^-1^ for the maximum force, 0.05 GPa for the maximum stress, 10^-5^ eV/atom for energy, and 2.0×10^-6^ eV/atom for self-consistent field tolerance. The Brillouin zone integration is performed using a 2×2×1 k-mesh. 15 Å vacuum space is implemented into the model to eliminate undesirable interactions between the bottom side of the slab and the molecules in the vacuum space.

When the optimization is completed, the density difference calculations are performed. Also, the diffusion barrier energy is located utilizing the well-known linear synchronous transit (LST) and quadratic synchronous transit (QST) methods.

**S2 Supplementary Figures and Tables**

**
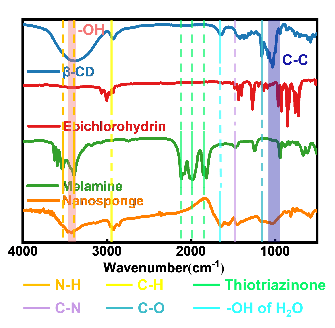
**

**Fig. S1** FTIR spectra of melamine, epichorohydrin, β-cyclodextrin, and cyclodextrin-based nanosponge

**Description:** The FTIR spectroscopy is employed to characterize the functional groups present in the polymer and NPCs. As shown in Fig. S1, the peak at 3419.57 cm^-1^ is attributed to the stretching vibration of -OH groups, while the peak at 2924.25 cm^-1^ is assigned to the stretching vibration of CH_2_ groups. The peak at 1645.57 cm^-1^ is considered the bending vibration absorption peak of -OH groups of water molecules adsorbed by β-cyclodextrin, serving as evidence for the presence of β-cyclodextrin [S7]. The peak at 1553.99 cm^-1^ is attributed to the in-plane stretching peak of C-N bonds, indicating the presence of melamine [S8]. Furthermore, compared to β-cyclodextrin, characteristic peaks of nanosponges exhibit certain shifts, and the intensity of the stretching vibration absorption peaks is weakened corresponding to C-O and C-C bonds at 1162 cm^-1^ and 1036.57 cm^-1^, respectively, suggesting cross-linking reactions of β-cyclodextrin [S7, S8].

**
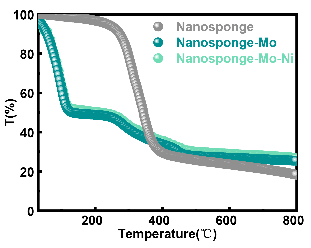
**

**Fig. S2** TGA curves of cyclodextrin-based nanosponge

**Description:** The TG curves are utilized for the preliminary determination of the initial carbonization temperature of nanosponge, as shown in Fig. S2. At the nitrogen atmosphere, the TG curve of nanosponge and its impregnated metal salt solution are mainly divided into three sections. The first stage involves the loss of free water and bound water in the polymer, primarily occurring between room temperature and 130°C. It is noteworthy that the weight loss rate of nanosponge impregnated with metal salt solution is significantly higher than that of nanosponge without impregnation, indicating the excellent water absorption capability of nano sponges. This heightened absorption could be attributed to the ability of nanosponge to adsorb some free and crystalline water within their pore structure, thereby enhancing the adsorption capacity of metal salt molecules on their surface ^[8]^. The second stage involves the breakage of polymer molecular chains, primarily occurring in the temperature range of 220 to 430 °C. Within this temperature range, functional groups within the polymer break down, accompanied by the loss of oxygen and hydrogen elements [S9, S10]. When the temperature exceeds 430 °C, this corresponds mainly to the formation of carbonaceous materials. Therefore, these results provide the theoretical and experimental supports to gain the targeted samples in our experimental conditions.

**
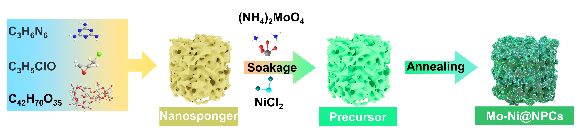
**

**Fig. S3** Schematic diagram of the process of NPCs and its derivatives

**
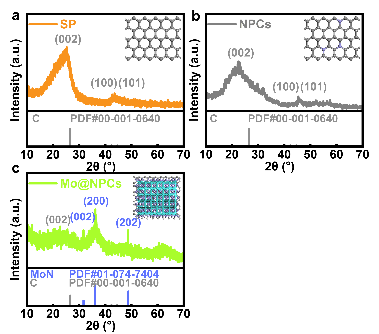
**

**Fig. S4** XRD patterns of (**a**) SP, (**b**) NPCs, and (**c**) Mo@NPCs

**
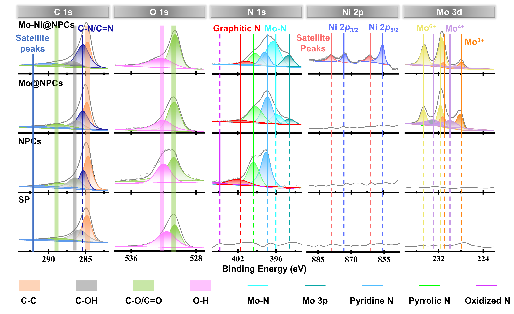
**

**Fig. S5** XPS spectra of Mo-Ni@NPCs, Mo@NPCs, NPCs, and SP

**
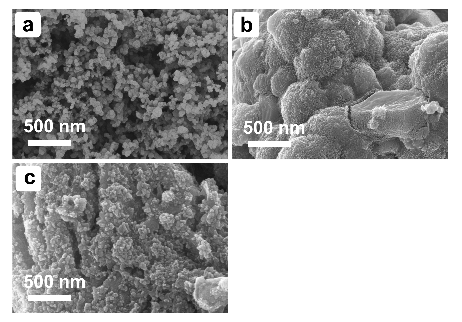
**

**Fig. S6** SEM images of (**a**) SP, (**b**) NPCs, and (**c**) Mo@NPCs

**
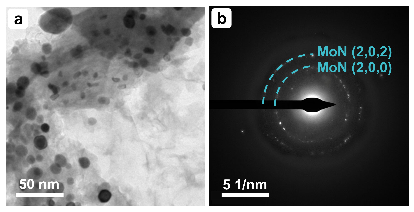
**

**Fig. S7** (**a**) HRTEM image and (**b**) SAED image of Mo@NPCs

**
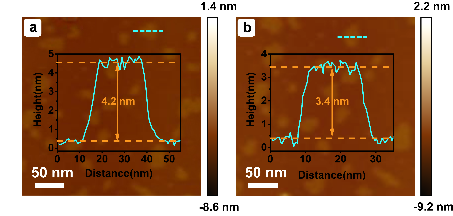
**

**Fig. S8** AFM images of (**a**) Mo@NPCs and (**b**) Mo-Ni@NPCs

**
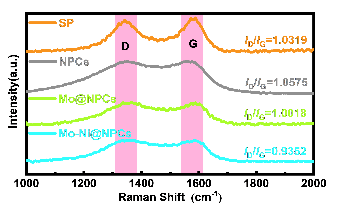
**

**Fig. S9** Raman spectra of Mo-Ni@NPCs, Mo@NPCs, NPCs, and SP

**
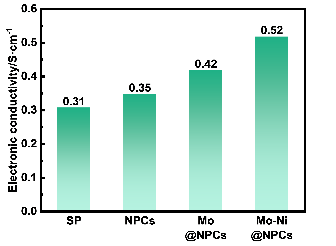
**

**Fig. S10** The electronic conductivities of Mo-Ni@NPCs, Mo@NPCs, NPCs, and SP

**
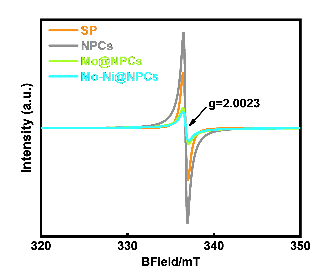
**

**Fig. S11** EPR spectra of Mo-Ni@NPCs, Mo@NPCs, NPCs, and SP (g=2.0023)

**
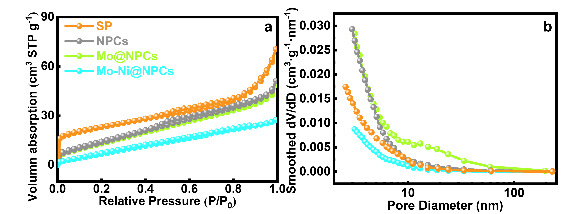
**

**Fig. S12** (**a**) N_2_ adsorption/desorption isothermal curves and (**b**) the pore size distributions of Mo-Ni@NPCs, Mo@NPCs, NPCs, and SP

**
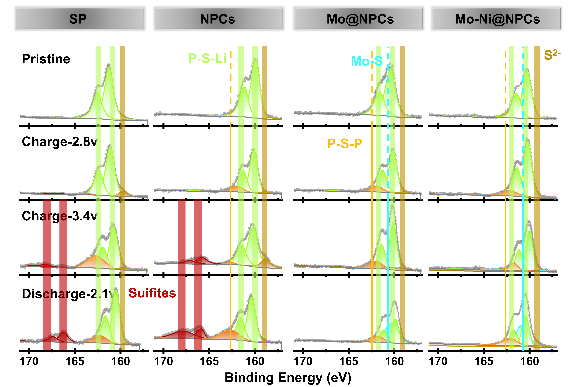
**

**Fig. S13** *ex-situ* XPS spectra of cathodes of SP/LPSC-based ASSLBs with non-AMs, NPCs/LPSC-based ASSLBs with non-AMs, Mo@NPCs/LPSC-based ASSLBs with non-AMs, and Mo-Ni@NPCs/LPSC-based ASSLBs with non-AMs at first cycle

**
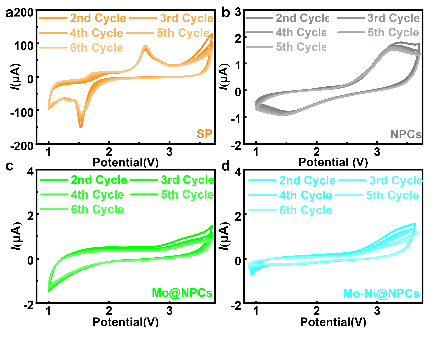
**

**Fig. S14** The 2^nd^-5^th^ CV curves of SP/LPSC-based ASSLBs with non-AMs, NPCs/LPSC-based ASSLBs with non-AMs, Mo@NPCs/LPSC-based ASSLBs with non-AMs, and Mo-Ni@NPCs/LPSC-based ASSLBs with non-AMs

**
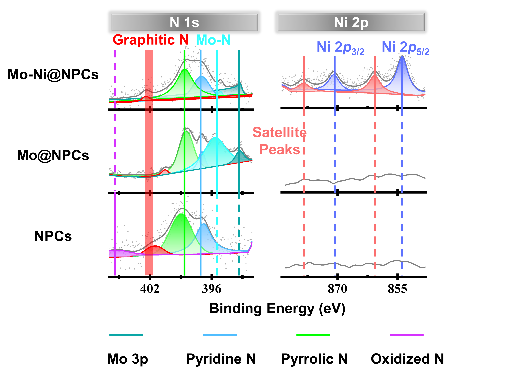
**

**Fig. S15** Mo3p XPS spectra and Ni2p XPS spectra of cathodes of NPCs/LPSC-based ASSLBs with non-AMs, Mo@NPCs/LPSC-based ASSLBs with non-AMs, and Mo-Ni@NPCs/LPSC-based ASSLBs with non-AMs

**
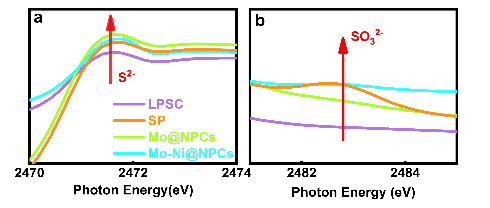
**

**Fig. S16** The enlarged views of (**a**) S^2-^ and (**b**) sulfites on XANES spectra of S K-edge of LPSC and the cathodes of SP/LPSC-based ASSLBs with non-AMs, Mo@NPCs/LPSC-based ASSLBs with non-AMs, Mo-Ni@NPCs/LPSC-based ASSLBs with non-AMs after 100 cycles (0.1C)

**
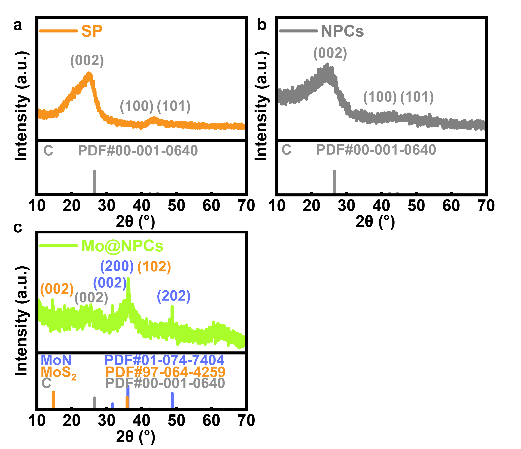
**

**Fig. S17** XRD patterns of (**a**) SP, (**b**) NPCs, and (**c**) Mo@NPCs after 1000 cycles

**
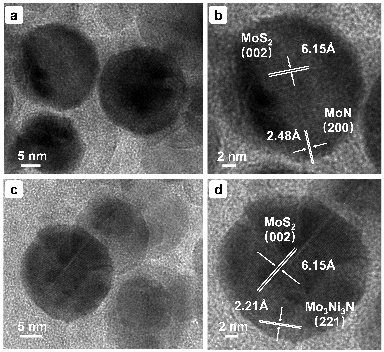
**

**Fig. S18** HRTEM images of (**a-b**) Mo@NPCs and (**c-d**) Mo-Ni@NPCs after 10 cycles

**
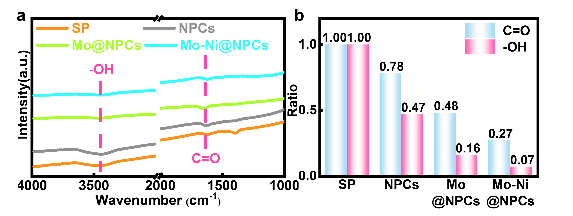
**

**Fig. S19** (**a**) FTIR spectra and (**b**) the relative content of functional groups of Mo-Ni@NPCs, Mo@NPCs, NPCs, and SP

**Description:** In order to quantitatively compare the contents of hydroxyl groups and carbonyl groups on SP, NPCs, Mo@NPCs and Ni-Mo@NPCs, the corresponding peaks areas SP are normalized to 1, while the peak area values of carbonyl species and hydroxyl species on other counterparts are obtained by dividing the peak area of their respective species by the corresponding peaks areas on SP.

**
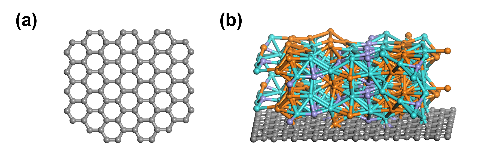
**

**Fig. S20** Structures of (**a**) SP and (**b**) Mo-Ni@NPCs

**
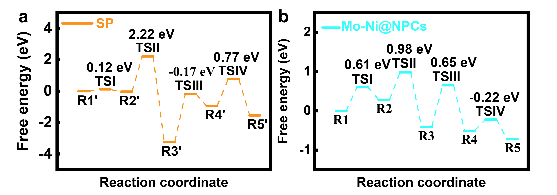
**

**Fig. S21** DFT-computed free energy diagrams of reaction pathway on (**a**) SP|LPSC interface and (**b**) Mo-Ni@NPCs|LPSC interface

**Description:** The interfacial reactions between different carbon materials and sulfide electrolytes are illustrated in Equations S1-S6.

Li_6_PS_5_Cl→Li_3_PS_4_+Li_2_S+LiCl (S1)

PS_4_^3-^+*OH→PS_4_^3-^-OH (S2)

PS_4_^3-^-*OH→PS_3_O^3-^-SH (S3)

PS_3_O^3-^-SH→PS_3_O^3-^+*SH (S4)

2Li_2_S+3*OH+*SH→Li_2_SO_3_+2H_2_S+2Li^+^+6e^-^ (S5)

Mo^3+^+3Li_2_S+2*SH→2MoS_2_+H_2_S+6Li^+^+5e^-^ (S6)

**
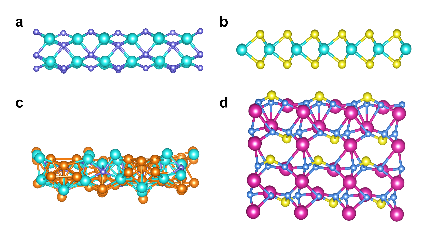
**

**Fig. S22** Structures of (**a**) MoN, (**b**) MoS_2_, (**c**) Mo_3_Ni_3_N, and (**d**) Li_2_SO_3_

**
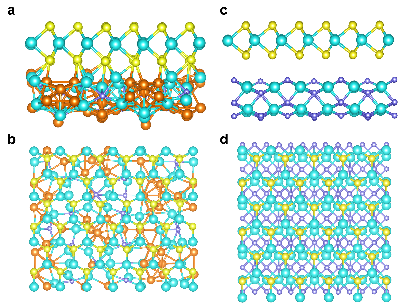
**

**Fig. S23** (**a, c**) The side views and (**b, d**) the top views of MoS_2_-Mo_3_Ni_3_N heterostructure and MoS_2_-MoN heterostructure

**
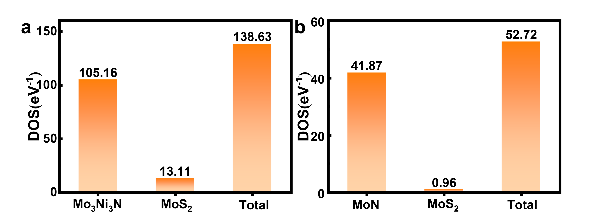
**

**Fig. S24** DOS at the Fermi level (E-E_f_=0) for (**a**) MoS_2_(001)/Mo_3_Ni_3_N(221) heterostructure and (**b**) MoS_2_(001)/MoN(200) heterostructure

**
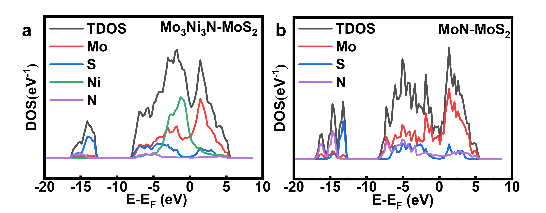
**

**Fig. S25** TDOS and PDOS of (**a**) MoS_2_-Mo_3_Ni_3_N heterostructure and (**b**) MoS_2_-MoN heterostructure

**
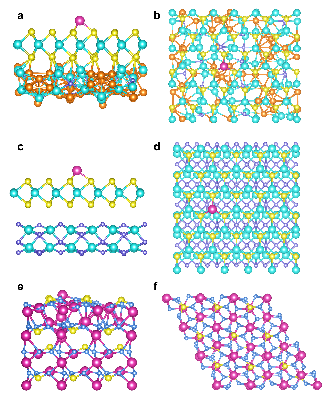
**

**Fig. S26** Adsorption states of Li^+^ ions on surfaces of MoS_2_-Ni_3_Mo_3_N heterostructure (**a**. side view, **b**. top view), MoS_2_-MoN heterostructure (**c**. side view, **d**. top view), and Li_2_SO_3_ (**e**. side view, **f**. top view)

**
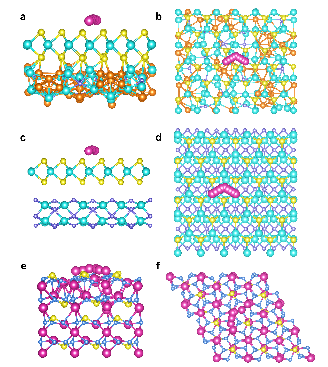
**

**Fig. S27** Migration pathways of Li^+^ ions on surfaces of MoS_2_-Ni_3_Mo_3_N heterostructure (**a**. side view, **b**. top view), MoS_2_-MoN heterostructure (**c**. side view, **d**. top view), and Li_2_SO_3_ (**e**. side view, **f**. top view)

**
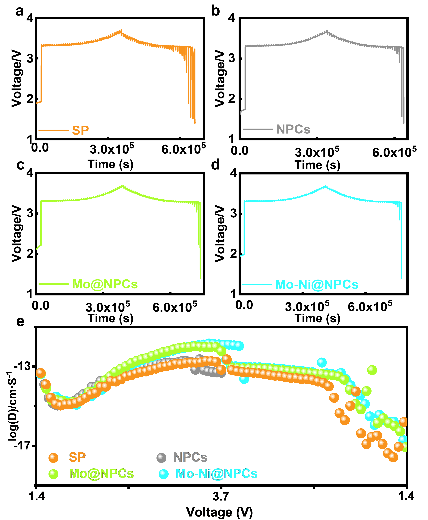
**

**Fig. S28** GITT curves of (**a**) SP/LCO/LPSC-based ASSLBs, (**b**) NPCs/LCO/LPSC-based ASSLBs, (**c**) Mo@NPCs/LCO/LPSC-based ASSLBs, and (**d**) Mo-Ni@NPCs /LCO/LPSC-based ASSLBs. (**e**) The Li^+^ ions diffusion coefficient of SP/LCO/LPSC-based ASSLBs, NPCs/LCO/LPSC-based ASSLBs, Mo@NPCs/LCO/LPSC-based ASSLBs, and Mo-Ni@NPCs/LCO/LPSC-based ASSLBs

**
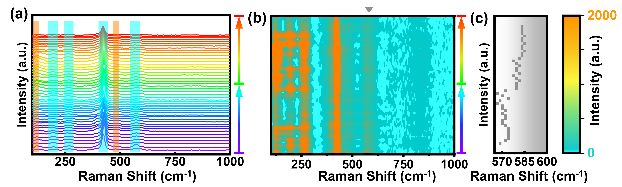
**

**Fig. S29** *operando* Raman spectra and the contour plots of cathodes of NPCs/LCO/LPSC-based ASSLBs

**
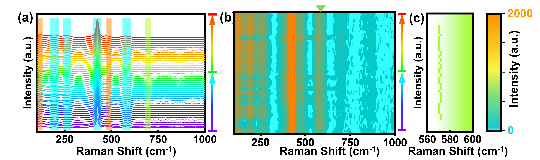
**

**Fig. S30** *operando* Raman spectra and the contour plots of cathodes of Mo@NPCs/LCO/LPSC-based ASSLBs


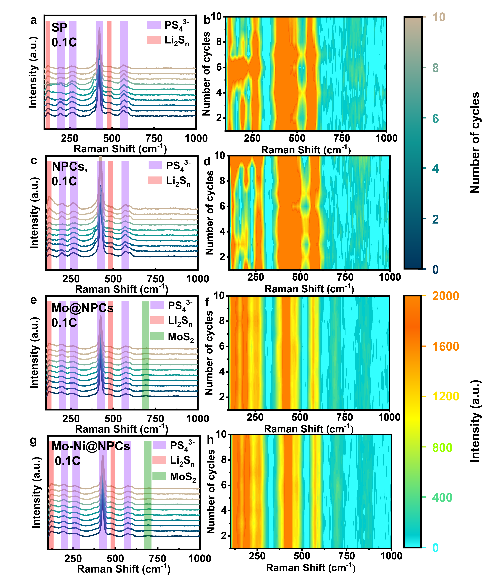


**Fig. S31** The *operando* Raman spectra and the contour plots of cathodes of (**a, b**) SP/LCO/LPSC-based ASSLBs, (**c, d**) NPCs/LCO/LPSC-based ASSLBs, (**e, f**) Mo@NPCs/LCO/LPSC-based ASSLBs, (g, h) Mo-Ni@NPCs/LCO/LPSC-based ASSLBs after different cycles

**
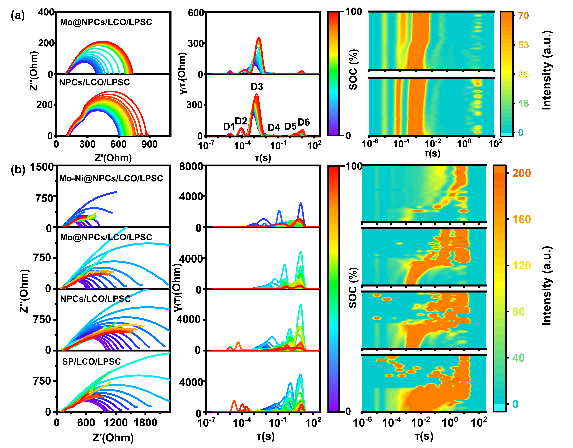
**

**Fig. S32** (**a**) The *in-situ* GEIS, the DRT analyses and the contour plots of DRT for NPCs/LCO/LPSC-based ASSLBs, and Mo@NPCs/LCO/LPSC-based ASSLBs at 1^st^ cycle during the charge process at 0.1C. (**b**) The *in-situ* GEIS, the DRT analyses and the contour plots of DRT for SP/LCO/LPSC-based ASSLBs, NPCs/LCO/LPSC-based ASSLBs, Mo@NPCs/LCO/LPSC-based ASSLBs, and Mo-Ni@NPCs/LCO/LPSC-based ASSLBs at 1^st^ cycle during the discharge process at 0.1C

**
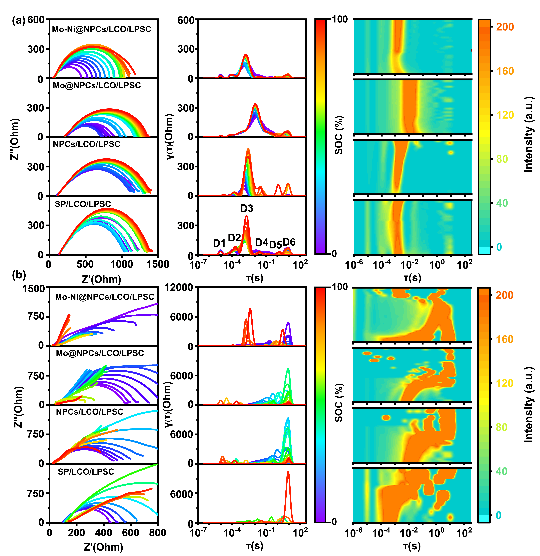
**

**Fig. S33** (**a**) *in-situ* GEIS, the DRT analyses and the contour plots of DRT for SP/LCO/LPSC-based ASSLBs, NPCs/LCO/LPSC-based ASSLBs, Mo@NPCs/LCO/LPSC-based ASSLBs, and Mo-Ni@NPCs/LCO/LPSC-based ASSLBs at 1^st^ cycle during the charge process at 0.2C. (**b**) The *in-situ* GEIS, the DRT analyses and the contour plots of DRT for SP/LCO/LPSC-based ASSLBs, NPCs/LCO/LPSC-based ASSLBs, Mo@NPCs/LCO/LPSC-based ASSLBs, and Mo-Ni@NPCs/LCO/LPSC-based ASSLBs at 1^st^ cycle during the discharge process at 0.2C

**
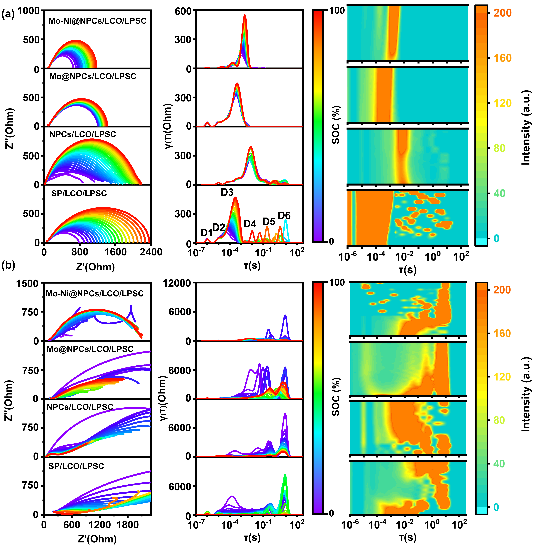
**

**Fig. S34** (**a**) The *in-situ* GEIS, the DRT analyses and the contour plots of DRT for SP/LCO/LPSC-based ASSLBs, NPCs/LCO/LPSC-based ASSLBs, Mo@NPCs/LCO/LPSC-based ASSLBs, and Mo-Ni@NPCs/LCO/LPSC-based ASSLBs at 1^st^ cycle during the charge process at 0.5C. (**b**) The *in-situ* GEIS, the DRT analyses and the contour plots of DRT for SP/LCO/LPSC-based ASSLBs, NPCs/LCO/LPSC-based ASSLBs, Mo@NPCs/LCO/LPSC-based ASSLBs, and Mo-Ni@NPCs/LCO/LPSC-based ASSLBs at 1^st^ cycle during the discharge process at 0.5C

**
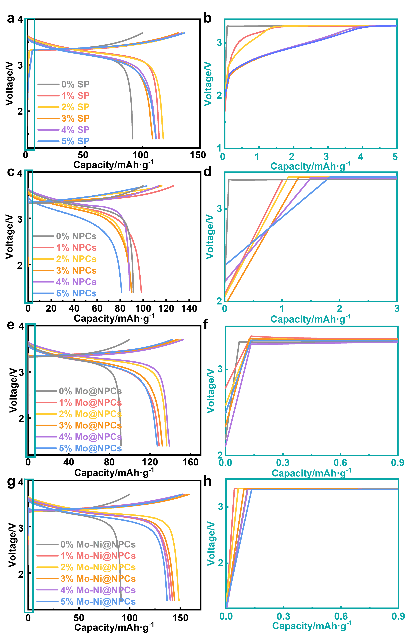
**

**Fig. S35** The effects of the content of (**a-b**) SP, (**c-d**) NPCs, (**e-f**) Mo@NPCs, and (**g-h**) Mo-Ni@NPCs on the 1^st^ cycle charge/discharge performances of ASSLBs (0.1C, RT)

**
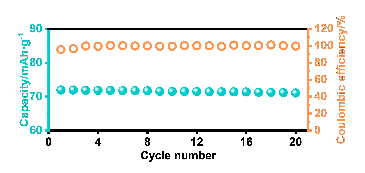
**

**Fig. S36** The long cycle stability of LCO/LPSC-based ASSLBs at room temperature (0.1C)

**
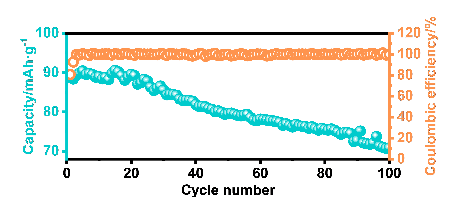
**

**Fig. S37** The long cycle stability of NPCs/LCO/LPSC-based ASSLBs at room temperature (0.1C)

**
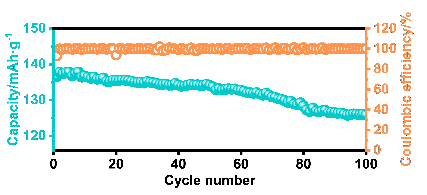
**

**Fig. S38** The long cycle stability of Mo@NPCs/LCO/LPSC-based ASSLBs at room temperature (0.1C)

**
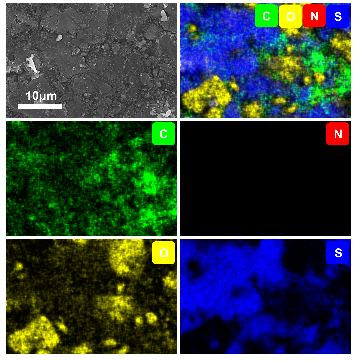
**

**Fig. S39** SEM images of cathodes of SP/LCO/LPSC-based ASSLBs before cycle

**
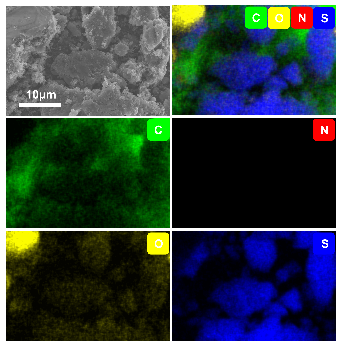
**

**Fig. S40** SEM images of cathodes of SP/LCO/LPSC-based ASSLBs after 100 cycles

**
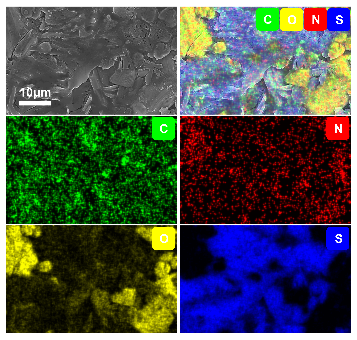
**

**Fig. S41** SEM images of cathodes of NPCs/LCO/LPSC-based ASSLBs before cycle

**
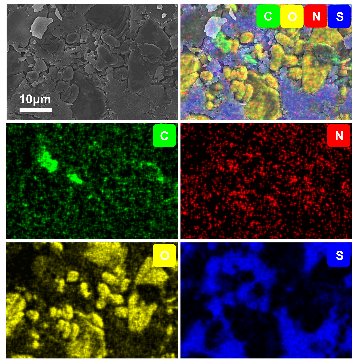
**

**Fig. 42** SEM images of cathodes of NPCs/LCO/LPSC-based ASSLBs after 100 cycles

**
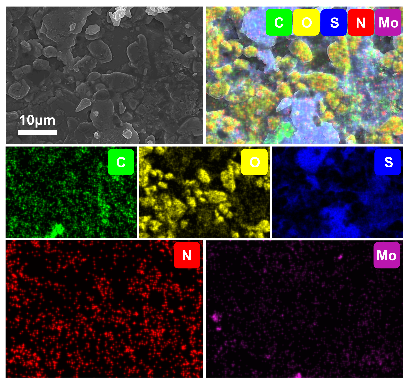
**

**Fig. S43 S**EM images of composite cathodes of Mo@NPCs/LCO/LPSC-based ASSLBs before cycle

**
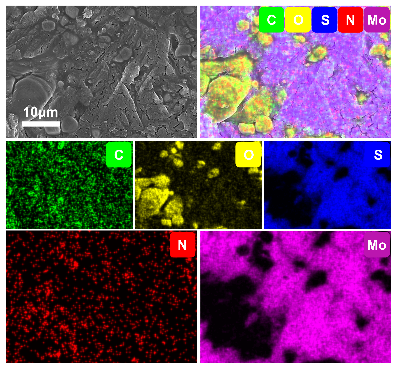
**

**Fig. S44** SEM images of composite cathodes of Mo@NPCs/LCO/LPSC-based ASSLBs after 100 cycles

**
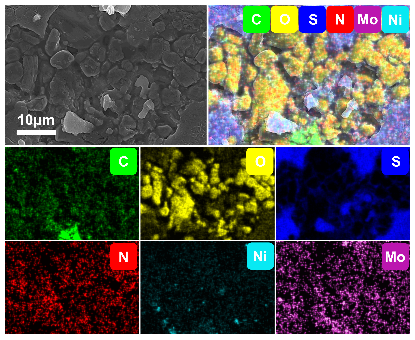
**

**Fig. S45** SEM images of composite cathodes of Mo-Ni@NPCs/LCO/LPSC-based ASSLBs before cycle

**
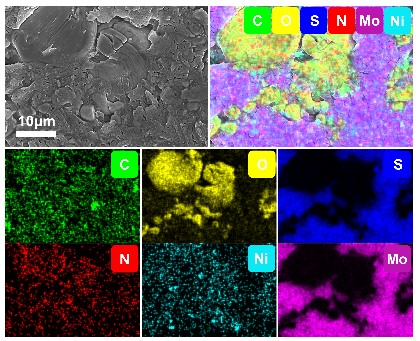
**

**Fig. S46** SEM images of composited cathodes of Mo-Ni@NPCs/LCO/LPSC-based ASSLBs after 100 cycles

**
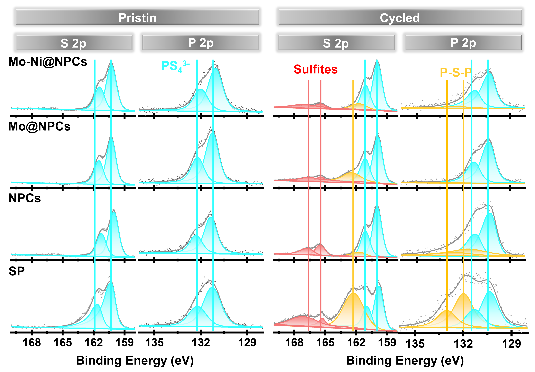
**

**Fig. S47** XPS spectra of S2p and P2p for cathodes of Mo-Ni@NPCs/LCO/LPSC-based ASSLBs, Mo@NPCs/LCO/LPSC-based ASSLBs, NPCs/LCO/LPSC-based ASSLBs and SP/LCO/LPSC-based ASSLBs before and after 100 cycles

**
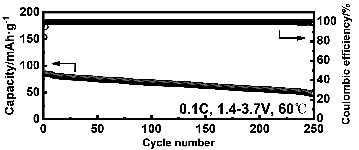
**

**Fig. S48** The long cycle stability of NPCs/LCO/LPSC-based ASSLBs at 60℃ (0.1C)**
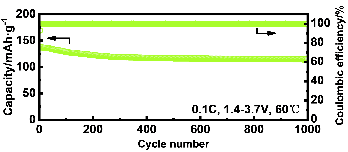
**

**Fig. S49** The long cycle stability of Mo@NPCs/LCO/LPSC-based ASSLBs at 60℃ (0.1C)

**
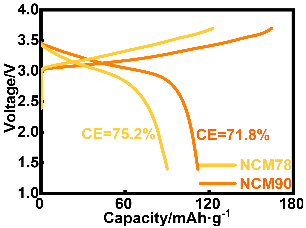
**

**Fig. S50** The 1^st^ cycle charge/discharge performances of SP/NCM/LPSC-based ASSLBs

**
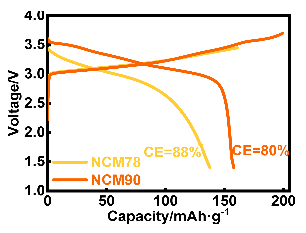
**

**Fig. S51** The 1^st^ cycle charge/discharge performances of Mo-Ni@NPCs/NCM/LPSC-based ASSLBs

**
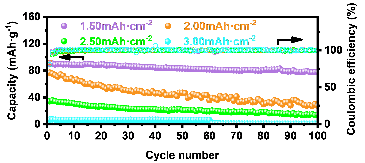
**

**Fig. S52** The effects of different areal capacities of LCO on long cycle stabilities of SP/LCO/LPSC-based ASSLBs

**
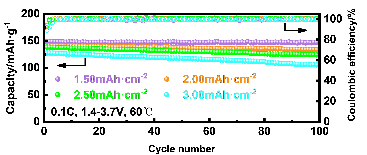
**

**Fig. S53** The effects of different areal capacities of LCO on long cycle stabilities of Mo-Ni@NPCs/LCO/LPSC-based ASSLBs

**
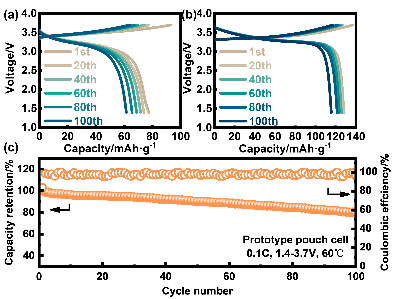
**

**Fig. S54** (**a**) The charge/discharge curves for 1.8mAh SP/LCO/LPSC-based prototype pouch cell at a 0.1 C/0.1 C charge/discharge rate plotted with selected cycle numbers. (**b**) The charge/discharge curves for 1.8mAh Mo-Ni@NPCs/LCO/LPSC-based prototype pouch cell at a 0.1 C/0.1 C charge/discharge rate plotted with selected cycle numbers. (**c**) The cycling performance and coulombic efficiency of SP/LCO/LPSC-based prototype pouch cell (1.8 mAh) are plotted against the cycle numbers. A constant current (CC) model with the charge/discharge rate of 0.1C/0.1C is applied (voltage windows, 1.4-3.7V vs. Li^+^/(In-InLi) at 60℃). The area capacity loading of the LCO cathodes is 0.15 mAh·cm^-2^ (0.1C=0.015mA·cm^-2^).

**Table S1** Specific surface area of SP, NPCs, Mo@NPCs, and Mo-Ni@NPCs

| **Sample** | **Specific surface area(m^2^·g^-1^)** |
| --- | --- |
| SP | 62.00 |
| NPCs | 53.59 |
| Mo@NPCs | 48.60 |
| Mo-Ni@NPCs | 20.39 |

**Table S2** Contents of different categories of C species from C1s XPS spectra in SP, NPCs, Mo@NPCs, and Mo-Ni@NPCs

|  | **SP** | **NPCs** | **Mo@NPCs** | **Mo-Ni@NPCs** |
| --- | --- | --- | --- | --- |
| C-C | 66.38 | 46.29 | 32.76 | 29.27 |
| C-N/C=N | / | 23.70 | 40.78 | 48.02 |
| C-OH | 12.79 | 16.26 | 10.98 | 8.70 |
| C-O/C=O | 7.48 | 10.46 | 10.69 | 2.78 |
| Satellite peaks | 13.35 | 3.29 | 4.79 | 11.23 |

**Table S3** Contents of different categories of O species from O1s XPS spectra in SP, NPCs, Mo@NPCs, and Mo-Ni@NPCs

|  | **SP** | **NPCs** | **Mo@NPCs** | **Mo-Ni@NPCs** |
| --- | --- | --- | --- | --- |
| C-O/C=O | 47.76 | 51.48 | 65.52 | 71.80 |
| O-H | 52.24 | 48.52 | 34.48 | 28.20 |

**Table S4** The lattice parameters and mismatches of MoS_2_-MoN heterostructure and MoS_2_-Mo_3_Ni_3_N heterostructure

| **Sample** | ***a*/nm** | ***b*/nm** | ***d*/nm** | **Mismatch/%** |
| --- | --- | --- | --- | --- |
| MoN | 0.5748 | 0.5648 | / | / |
| Mo_3_Ni_3_N | 0.6635 | 0.6668 | / | / |
| MoS_2_ | 0.3170 | 1.8340 | / | / |
| MoS_2_-MoN | 16.4097 | 16.7561 | 4.92867 | 1.5000 |
| MoS_2_-Mo_3_Ni_3_N | 18.7165 | 15.9701 | 4.95722 | 1.2000 |

**Table S5** The effects of CCAs contents in composite cathodes for capacities and coulomb efficiencies of ASSLBs (0.1C, RT)

| **Sample** | **Content/%** | **Capacity/mAh·g^-1^** | **Coulomb efficiency/%** |
| --- | --- | --- | --- |
| SP | 1 | 114.49 | 83.78 |
|  | 2 | 118.13 | 84.38 |
|  | 3 | 108.91 | 83.13 |
|  | 4 | 111.87 | 83.52 |
|  | 5 | 111.58 | 81.84 |
| NPCs | 1 | 98.45 | 78.09 |
|  | 2 | 89.88 | 77.30 |
|  | 3 | 90.09 | 78.58 |
|  | 4 | 88.32 | 77.93 |
|  | 5 | 81.28 | 78.78 |
| Mo@NPCs | 1 | 128.98 | 89.45 |
|  | 2 | 136.59 | 90.57 |
|  | 3 | 132.25 | 90.24 |
|  | 4 | 139.27 | 90.08 |
|  | 5 | 127.31 | 89.75 |
| Mo-Ni@NPCs | 1 | 142.29 | 91.26 |
|  | 2 | 145.92 | 93.26 |
|  | 3 | 143.27 | 91.08 |
|  | 4 | 140.47 | 92.05 |
|  | 5 | 136.95 | 90.36 |

**Table S6** The performance in previously reported representative literatures compared with our work

| **Number** | **SSEs**  **system** | **Cathode**  **structure** | **1^st^ CE/%** | **Capacity retention at 100 cycles/%** | **Areal capacity/**  **mAh·cm^-2^** | **Reference** |
| --- | --- | --- | --- | --- | --- | --- |
| 1 | LPSC | NCM/VG-CNF/LPSC | 83.45 | 85.52 | 1.43 | [S2] |
| 2 | LPS | NCM/VGCF/LPS | 65.71 | 54.00 | 2.15 | [S11] |
| 3 | LGPS | NCM@LCO@LNO/LGPS | 84.21 | 68.80 | 1.84 | [S12] |
| 4 | LSPSC | SC-NCM/LSPSC | 85.71 | 63.00 | 2.45 | [S13] |
| 5 | LPSC | S-KB-LiTFSI/CNTs/LPSC | 79.02 | 78.00 | 1.15 | [S14] |
| 6 | LPSC | LiNbO_3_@NCM/CB/LPSC | 73.08 | 83.42 | 1.65 | [S15] |
| 7 | Li_2_S-P_2_S_5_ | NCM/ Denka black /Li_2_S-P_2_S_5_ | 88.03 | 88.52 | 1.42 | [S16] |
| 8 | LGPS | NCM/CNTs/LGPS | 79.82 | 60.40 | 1.34 | [S17] |
| 9 | LPSC | NCM/SP/LPSC | 82.51 | 63.40 | 1.21 | [S18] |
| 10 | LPSC | NCM/PTFE/LPSC | 67.10 | 85.24 | 1.40 | [S19] |
| 11 | LSPSC | NCM/LSPSC | 84.29 | 79.40 | 2.20 | [S20] |
| 12 | LPSC | NCM/LPSC | 68.80 | 83.30 | 2.10 | [S21] |
| 13 | β-LPS | NCM/VGCF/β-LPS | 79.80 | 85.71 | 2.00 | [S22] |
| 14 | LGPS | CS-NCA@LiNbO_3_/LGPS | 82.90 | 88.64 | 2.52 | [S23] |
| 15 | LPSC | Li_4_C_8_H_2_O_6_/SP/LPSC | 83.30 | 82.60 | 1.93 | [S24] |
| 16 | LGPS | LNMO/AB/LGPS | 75.60 | 52.90 | 1.77 | [S25] |
| 17 | LPSC | NMC/RGO/LPSC | 74.90 | 52.10 | 1.35 | [S26] |
| 18 | LPSC | NCM/SC-Gr/LPSC | 78.80 | 74.40 | 1.35 | [S27] |
| 19 | LPSC | Ti_3_(PO_4_)_4_-NCM/LPSC | 74.58 | 64.86 | 1.76 | [S28] |
| 20 | LIC | LRMO/SBS/SP/LIC | 73.63 | 79.84 | 1.15 | [S29] |
| 21 | LGPS | NCM/LGPS | 84.30 | 79.04 | 2.41 | [S30] |
| 22 | LPSC | NCM/CNF/LPSC | 75.90 | 77.78 | 1.91 | [S31] |
| 23 | LPSC | NCM/SP/LPSC | 83.59 | 76.60 | 1.61 | [S32] |
| 24 | LGPS | LCO-NiS-CNT@LPS/LSPS | 84.21 | 71.84 | 0.34 | [S33] |
| **25** | **LPSC** | **LCO/Mo-Ni@NPCs/LPSC** | **94.01** | **96.88** | **1.50** | **This Work** |
| **26** |  |  | **93.26** | **94.63** | **2.00** |  |
| **27** |  |  | **91.52** | **89.36** | **2.50** |  |
| **28** |  |  | **89.28** | **82.59** | **3.00** |  |

**Supplementary References**

1. Y. Chen, W. Li, C. Sun, J. Jin, Q. Wang et al., Sustained release-driven formation of ultrastable SEI between Li_6_PS_5_Cl and lithium anode for sulfide-based solid-state batteries. Adv. Energy Mater. **11**(4), 2002545 (2021). https://doi.org/10.1002/aenm.202002545
2. F. Hippauf, B. Schumm, S. Doerfler, H. Althues, S. Fujiki et al., Overcoming binder limitations of sheet-type solid-state cathodes using a solvent-free dry-film approach. Energy Storage Mater. **21**, 390-398 (2019). https://doi.org/10.1016/j.ensm.2019.05.033
3. Y. Lee, S. Fujiki, C. Jung, N. Suzuki, N Yashiro et al., High-energy long-cycling all-solid-state lithium metal batteries enabled by silver-carbon composite anodes. Nat. Energy **5**, 299-308 (2020). https://doi.org/10.1038/s41560-020-0575-z
4. G. Kresse, D. Joubert, From ultrasoft pseudopotentials to the projector augmented-wave method. Phys. Rev. B **59**(3), 1758 (1999). https://doi.org/10.1103/PhysRevB.59.1758
5. J. P. Perdew, K. Burke, M. Ernzerhof, Generalized gradient approximation made simple. Phys. Rev. Lett. **77**(18), 3865 (1996). https://doi.org/10.1103/PhysRevLett.77.3865
6. S. Grimme, J. Antony, S. Ehrlich, H. Krieg, A consistent and accurate ab initio parametrization of density functional dispersion correction (DFT-D) for the 94 elements H-Pu. J. Chem. Phys. **132**(15), 154104 (2010). https://doi.org/10.1063/1.3382344
7. S. Iravani, R. Varma, Nanosponges for Water Treatment, Progress and Challenges. Appl. Sci. **12**(9), 4182 (2022). https://doi.org/10.3390/app12094182
8. H. Zhong, X. Gao, Z. Qiu, B. Sun, W. Huang et al., Insight into β-cyclodextrin polymer microsphere as a potential filtration reducer in water-based drilling fluids for high temperature application. Carbohydr. Polym. **249**, 116833 (2020). https://doi.org/10.1016/j.carbpol.2020.116833
9. H. Zhong, X. Gao, Z. Qiu, C. Zhao, X. Zhang et al., Formulation and evaluation of β-cyclodextrin polymer microspheres for improved HTHP filtration control in water-based drilling fluids. J. Mol. Liq. **313**, 223549 (2020). https://doi.org/10.1016/j.molliq.2020.113549
10. Y. Cui, Y. Wang, Z. Shao, A. Mao, W. Gao et al., Smart Sponge for Fast Liquid Absorption and Thermal Responsive Self-Squeezing. Adv. Mater. **32**(14), 1908249 (2020). https://doi.org/10.1002/adma.201908249
11. R. S. Negi, Y. Yusim, R. Pan, S. Ahmed, K. Volz et al., A Dry-Processed Al_2_O_3_/LiAlO_2_ Coating for Stabilizing the Cathode/Electrolyte Interface in High-Ni NCM-Based All-Solid-State Batteries. Adv. Mater. Interfaces **9**, 2101428 (2022). https://doi.org/10.1002/admi.202101428
12. 12 X. Li, Q. Sun, Z. Wang, D. Song, H. Zhang et al., Outstanding electrochemical performances of the all-solid-state lithium battery using Ni-rich layered oxide cathode and sulfide electrolyte. J. Power Sources **456**, 227997 (2020). https://doi.org/10.1016/j.jpowsour.2020.227997
13. X. Li, W. Peng, R. Tian, D. Song, Z. Wang et al., Excellent performance single-crystal NCM cathode under high mass loading for all-solid-state lithium batteries. Electrochim. Acta **363**, 137185 (2020). https://doi.org/10.1016/j.electacta.2020.137185
14. L. Hu, T. Yang, X. Yan, Y. Liu, W. Zhang et al., In situ construction of LiF-Li_3_N-Rich interface contributed to fast ion diffusion in all-solid-state lithium-sulfur batteries. ACS Nano **18**, 8463-8474 (2024). https://doi.org/10.1021/acsnano.4c00267
15. J. Zhang, H. Zhong, C. Zheng, Y. Xia, C. Liang et al., All-solid-state batteries with slurry coated LiNi_0.8_Co_0.1_Mn_0.1_O_2_ composite cathode and Li_6_PS_5_Cl electrolyte, Effect of binder content. J. Power Sources 391, 73-79 (2018). https://doi.org/10.1016/j.jpowsour.2018.04.069
16. K. Lee, S. Kim, J. Park, S. Park, A. Coskun et al., Selection of binder and solvent for solution-processed all-solid-state battery. J. Electrochem. Soc. **164**, A2075 (2017). https://doi.org/10.1149/2.1341709jes
17. S. Deng, Y. Sun, X. Li, Z. Ren, J. Liang et al., Eliminating the detrimental effects of conductive agents in sulfide-based solid-state batteries. ACS Energy Lett. **5**, 1243-1251 (2020). https://doi.org/10.1021/acsenergylett.0c00256
18. S. Park, G. Oh, J. Park, Y. Ha, S. Lee et al., Graphitic hollow nanocarbon as a promising conducting agent for solid-state lithium batteries. Small **15**, 1900235 (2019). https://doi.org/10.1002/smll.201900235
19. Z. Zhang, L. Wu, D. Zhou, W. Wang, X. Yao, Flexible sulfide electrolyte thin membrane with ultrahigh ionic conductivity for all-solid-state lithium batteries. Nano Lett. **21**(12), 5233-5239 (2021). https://doi.org/10.1021/acs.nanolett.1c01344
20. W. Jiang, X. Zhu, R. Huang, S. Zhao, X. Fan et al., Revealing the design principles of Ni-rich cathodes for all-solid-state batteries. Adv. Energy Mater. **12**, 2103473 (2022). https://doi.org/10.1002/aenm.202103473
21. F. Strauss, D. Stepien, J. Maibach, L. Pfaffmann, S. Indris et al., Influence of electronically conductive additives on the cycling performance of argyrodite-based all-solid-state batteries. RSC Adv. **10**, 1114-1119 (2020). https://doi.org/10.1039/C9RA10253A
22. F. Walther, S. Randau, Y. Schneider, J. Sann, M. Rohnke et al., Influence of carbon additives on the decomposition pathways in cathodes of lithium thiophosphate-based all-solid-state batteries. Chem. Mater. **32**, 6123-6136 (2020). https://doi.org/10.1021/acs.chemmater.0c01825
23. X. Li, M. Liang, J. Sheng, D. Song, H. Zhang et al., Constructing double buffer layers to boost electrochemical performances of NCA cathode for ASSLB. Energy Storage Mater. **18**, 100-106 (2019). https://doi.org/10.1016/j.ensm.2018.10.003
24. F. Song, Z. Wang, G. Sun, T. Ma, D. Wu et al., In-situ CNT-loaded organic cathodes for sulfide all-solid-state Li metal batteries. eTransportation, **18**, 100261 (2023). https://doi.org/10.1016/j.etran.2023.100261
25. G. Oh, M. Hirayama, O. Kwon, K. Suzuki, R. Kanno, Bulk-type all solid-state batteries with 5 V Class LiNi_0.5_Mn_1.5_O_4_ cathode and Li_10_GeP_2_S_12_ solid electrolyte. Chem. Mater. **28**, 2634-2640 (2016). https://doi.org/10.1021/acs.chemmater.5b04940
26. Y. Byeon, S. Yang, G. Yang, D. Kim, V. Avvaru et al., Conductive carbon embedded beneath cathode active material for longevity of solid-state batteries. J. Mater. Chem. A **12**, 8359-8369 (2024). https://doi.org/10.1039/D4TA00674G
27. J. Choi, K. Ko, S. Won, K. Saqib, T. Embleton et al., Important consideration for interface engineering of carbon-based materials in sulfide all-solid lithium-ion batteries. Energy Storage Mater. **71**, 103653 (2024). https://doi.org/10.1016/j.ensm.2024.103653
28. X. Wang, M. Zhou, Y. Deng, Z. Liu, H. Dong et al., Dual functional Ti_3_(PO_4_)_4_-coated NCM811 cathode enables highly stable sulfide-based all-solid-state lithium batteries. Chin. Chem. Lett. , 110307 (2024). https://doi.org/10.1016/j.cclet.2024.110307
29. X. Li, Q. Ye, Z. Wu, W. Zhang, H. Huang et al., High-voltage all-solid-state lithium batteries with Li_3_InCl_6_ electrolyte and LiNbO_3_ coated lithium-rich manganese oxide cathode. Electrochim. Acta **453**, 142361 (2023). https://doi.org/10.1016/j.electacta.2023.142361
30. G. Huang, Y. Zhong, X. Xia, X. Wang, C. Gu et al., Surface-modified and sulfide electrolyte-infiltrated LiNi_0.6_Co_0.2_Mn_0.2_O_2_ cathode for all-solid-state lithium batteries. J. Colloid Interface Sci. **632**, 11-18 (2023). <https://doi.org/10.1016/j.jcis.2022.11.048>
31. T. Embleton, J. Yun, J. Choi, J. Kim, K. Ko et al., Lithium-enhanced functionalized carbon nanofibers as a mixed electronic/ionic conductor for sulfide all solid-state batteries. Appl. Surf. Sci. **610**, 155490 (2023). <https://doi.org/10.1016/j.apsusc.2022.155490>
32. J. Cho, R. Rajagopal, D. Yoon, Y. Park, K. Ryu, Control of side reactions using LiNbO_3_ mixed/doped solid electrolyte for enhanced sulfide-based all-solid-state batteries. Chem. Eng. J. **452**, 138955 (2023). https://doi.org/10.1016/j.cej.2022.138955
33. M. Jiang, W. Fan, G. Liu, W. Weng, L. Cai et al., One‐dimensional NiS‐CNT@Li_7_P_3_S_11_ nanocomposites as ionic/electronic additives for LiCoO_2_ based all‐solid‐state lithium batteries. Electrochim. Acta **398**, 139230 (2021). https://doi.org/10.1016/j.electacta.2021.139280
